# Spatial-temporal distribution of human brucellosis in mainland China from 2004 to 2017 and an analysis of social and environmental factors

**DOI:** 10.1186/s12199-019-0839-z

**Published:** 2020-01-02

**Authors:** Cheng Peng, Yan-Jun Li, De-Sheng Huang, Peng Guan

**Affiliations:** 1 Department of Epidemiology, School of Public Health, China Medical University, Shenyang, 110122 China; 2 Department of Mathematics, School of Fundamental Sciences, China Medical University, Shenyang, 110122 China

**Keywords:** Human brucellosis, Spatial-temporal distribution, Cluster analysis

## Abstract

**Background:**

This study aimed to describe the changing distribution of human brucellosis between 2004 and 2017 in mainland China and seek scientific evidence of the relationship between socio-economic, environmental, and ecological factors and human brucellosis incidence.

**Methods:**

The annual numbers of brucellosis cases and incidence rates from 31 provinces in mainland China between 2004 and 2017 were obtained from the Data-Center for China Public Health Science. The number of monthly brucellosis cases in 2018 was obtained from the Chinese Center for Disease Control and Prevention. The electronic map of the People’s Republic of China was downloaded from the National Earth System Science Data Sharing Platform. Human population density, gross domestic product (GDP), and an inventory of cattle and sheep at the end of each year from 2004 to 2017 were obtained from the National Bureau of Statistics of China. Annual rainfall data from 31 provinces in the People’s Republic of China from 2004 to 2017 were collected from the China Meteorological Data Service Center. The risk distribution and changing trends of human brucellosis were mapped with ArcGIS. A cluster analysis was employed to identify geographical areas and periods with statistically significant incidence rates. Multivariate linear regression was used to determine possible factors that were significantly correlated with the presence of human brucellosis cases.

**Results:**

Human brucellosis cases have spread throughout the whole country. Human brucellosis cases occurred mostly from March to August and were concentrated from April to July. The inventory of sheep, GDP, and climate were significantly correlated with the presence of brucellosis cases in mainland China.

**Conclusions:**

The geographical expansion of human brucellosis in mainland China was observed, so did the high-incidence clusters between 2004 and 2017. Most of the cases were reported during the early spring to early summer (February–August). Results from the multivariate linear regression suggested that the inventory of sheep, GDP, and climate were significantly associated with the incidence of human brucellosis in mainland China.

## Background

Brucellosis, caused by *Brucella* species, has been recognized as one of the most common worldwide zoonotic diseases [1]. It not only causes substantial direct economic losses in animal production but also presents a great threat to human health [2]. In many countries, there has been a marked decrease in the incidence of human brucellosis, which can be attributed to successful domestic animal brucellosis control or eradication programs [3].

However, brucellosis is endemic in China [4, 5]. Human brucellosis remains a serious public health concern in China, with a 7.8% annual increase in the number of reported cases from 2007 to 2017 and more widespread natural foci [6, 7]. From the perspective of geographical distribution, the affected regions in China gradually expanded from the northern traditional pasturing regions to the agricultural areas and finally to the southern coastal and southwestern areas [7]. From the perspective of occupations at risk, human brucellosis cases have been identified in all classifications of occupations listed in China’s National Noticeable Infectious Disease Reporting (NIDR) system; notably, brucellosis cases have been reported in students, children, and retirees who were not directly exposed to livestock [8]. Low and insufficient awareness and knowledge about brucellosis have also been reported among the Chinese population [9, 10]. The abovementioned changing epidemiology of human brucellosis requires additional focus on the spatial-temporal distribution over time and a better understanding of potential drivers or socio-economic predictors to adjust brucellosis control strategies and allocate health resources.

Thus, the present study was conducted to visualize the annual and monthly human brucellosis incidence rates of each province in the People’s Republic of China from 2004 to 2017 and to explore the impacts of the gross domestic product (GDP), the number of cattle and sheep, and precipitation on the human brucellosis incidence rates in each province from 2004 to 2017, with the aim of providing information for the prevention and control of human brucellosis.

## Methods

### Data source

**Fig. 1:**
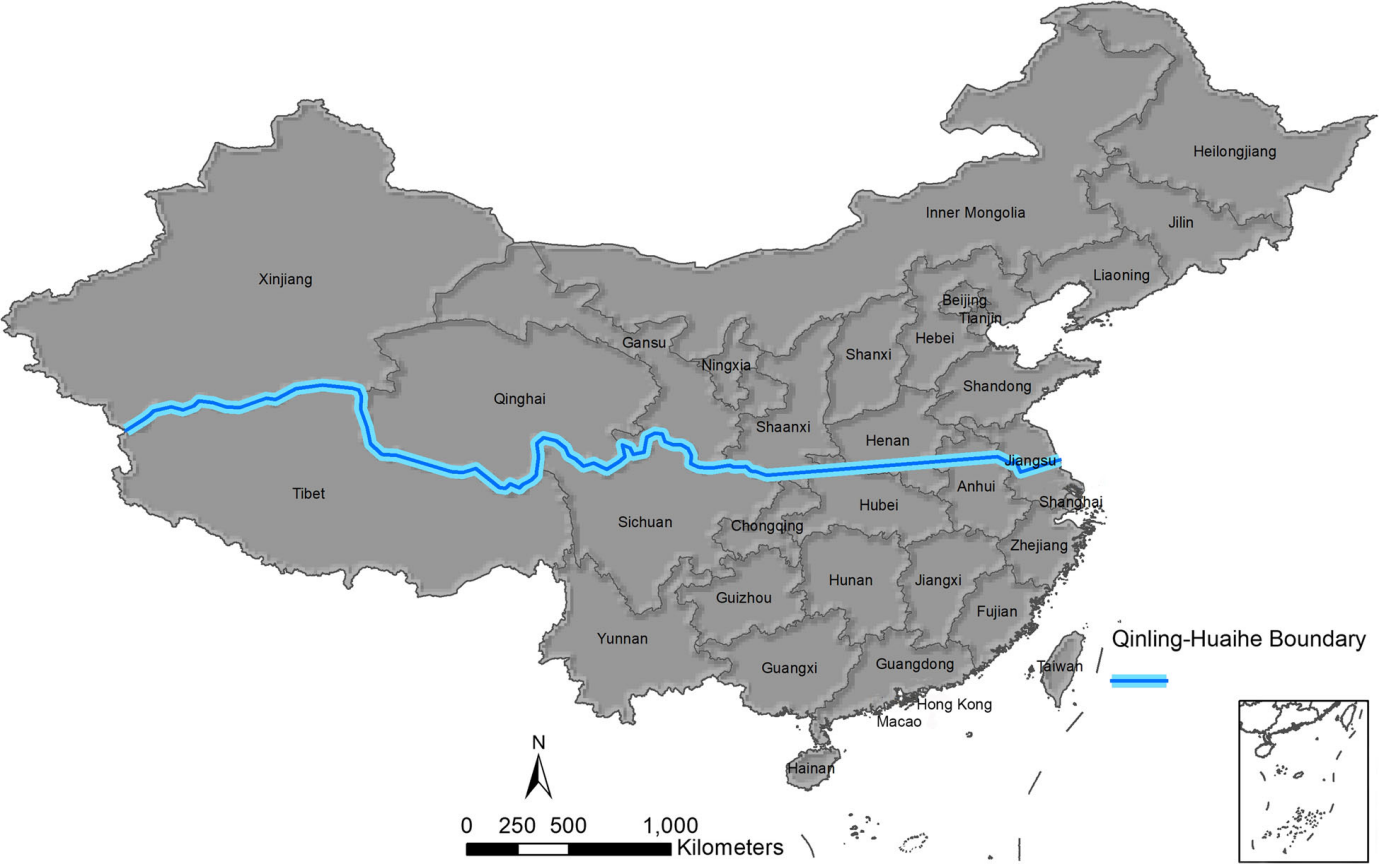
Geographical map of mainland China. Provinces were characterized as southern and northern regions according to the Qinling-Huaihe boundary. Areas south of the Qinling-Huaihe boundary are considered southern China, and areas north of the boundary are considered northern China

In this study, “province” in China was selected as the research unit, and the provinces were divided into two parts, southern and northern provinces, along the Qinling Mountains-Huaihe River line [7]. The map of China showing the province names and the division line is presented in Fig. 1. The annual numbers of brucellosis cases and incidence rates from 31 provinces in China between January 1, 2004, and December 31, 2017, were obtained from the Data-Center for China Public Health Science (http://www.phsciencedata.cn/Share/en/index.jsp). The numbers of monthly brucellosis cases between January 1, 2018, and December 31, 2018, were obtained from the Chinese Center for Disease Control and Prevention (http://www.chinacdc.cn/). An electronic map of China (1:1,000,000) was downloaded from the National Earth System Science Data Sharing Platform (http://www.geodata.cn). The information was unavailable regarding brucellosis incidence in Hong Kong, Macao, and Taiwan; we marked “Data unavailable” when necessary in the figures presented in this study.

Socio-economic, environmental, and ecological changes have been regarded as triggers for the emergence of emerging infectious diseases (EIDs) [11–13]. Pathogens have been indicated to be well adapted to and good at exploiting any social and environmental changes, such as the changes in human demographics or climate, resulting in new chances to spread [14]. In the present study, six factors were selected from the socio-economic (human population density and GDP), environmental (precipitation and climate type), and ecological (the inventory of cattle and the inventory of sheep) perspectives. The human population density in urban areas (persons per km^2^), GDP, and an inventory of cattle and sheep at the end of each year from 2004 to 2017 were obtained from National Bureau of Statistics of China (http://data.stats.gov.cn/). Annual rainfall data from 173 surface meteorological observation stations in 31 provinces in China (Hong Kong, Macao, and Taiwan were not included) from 2004 to 2017 (average rainfall per year, mm) were collected from China Meteorological Data Service Center (CMDC, http://data.cma.cn/en). The classification of climate type (tropical, subtropical, warm-temperate, mid-temperature, and cold areas) for each province was on the basis of a previous publication [7].

### Statistical analysis

The spatial distribution and temporal trend of brucellosis incidence and the relationship between the targeted risk factors and the number of brucellosis cases were explored and visualized using ArcGIS10.6 software (ESRI, Redlands, CA, USA). For the spatial analysis, the data existed in the form of discrete points, and there were no values for the unsampled points. Inversed distance weighted (IDW) interpolation estimates the unknown values by assuming that the weights of the unknown points are greater when they are closer to the known points. Interpolation generates a continuous surface of values. Inversed distance weighted (IDW) interpolation was performed and visualized based on the average incidence between 2004 and 2017 for each province.

Statistically significant high-incidence clusters (hot spots) and low-incidence clusters (cold spots) were identified and visualized with optimized hot spot analysis using ArcGIS 10.6 software. Spatial and temporal statistics were adopted to detect the geographical and temporal clusters between 2004 and 2017 using SaTScan version 9.6 (https://www.satscan.org/), and the results were visualized by ArcGIS10.6. With the assumption that brucellosis incidence followed a discrete Poisson distribution, the maximum spatial cluster size was set to 10% of the population at risk, the minimum temporal cluster size was set to 1 month, and the maximum temporal cluster size was set to 50% of the study period in the Poisson model. The standard Monte Carlo method was employed to determine the *P* value, and the number of Monte Carlo replications was 999. The most likely cluster, secondary likely clusters, and so on were identified by the parameter log-likelihood ratio (LLR). The LLR compares the difference in the number of cases inside and outside the cluster. The most likely cluster has the greatest value of LLR. Following the most likely cluster, the secondary clusters are outlined by the value of each LLR. A disease cluster was defined when the actual number of cases was greater than the expected number. Assuming that the portion of disease cases inside the scanned cluster was the same as the average portion throughout the whole area, we calculated the value of expected cases as the product of the population of the scanned cluster and the average incidence in the whole country.

Multivariate linear regression was conducted to investigate the association between the inventory of cattle, the inventory of sheep, GDP, human population density, precipitation, climate type, and brucellosis incidence between 2004 and 2017. The model was trained using the annual incidence, number of cattle, number of sheep, GDP, human population density, precipitation, and climate type from 2004 to 2017 for each province. The dataset was shuffled and split into training and testing sets at a ratio of 7 to 3. Before conducting the regression analysis, the values of the GDP, population density, and human brucellosis incidence were log-transformed. An adjusted *R*^2^ analysis was selected to evaluate the model performance, and the validation of the model was examined by linearity, a P-P plot, the variance inflation factor (VIF), and a residual versus fitted value scatter plot. A P-P plot was used to check whether the variables in the model were multivariate and normal. The VIF was employed to detect the scale of multicollinearity. A VIF value between 1 and 5 indicated moderate correlation; if the VIF had a value of 10 or more, the correlation was very high, and the model required further manipulation. Multilinear regression was conducted using Python 3.7 software (https://www.python.org/). The correlation coefficients of the chosen variables were obtained through multilinear regression, and we calculated the standardized partial regression coefficients to compare the contribution of each variable to the model.

## Results

### Brucellosis incidence in China

**Fig. 2:**
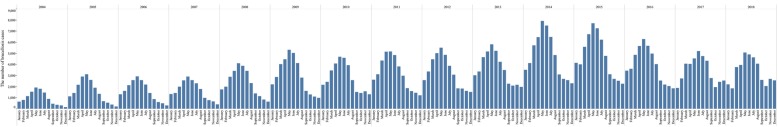
The number of monthly brucellosis cases from January 2004 to December 2018 in mainland China. The number of brucellosis patients increased between 2004 and 2018. Brucellosis occurred mostly from March to August and was concentrated in April to July in each year

**Fig. 3:**
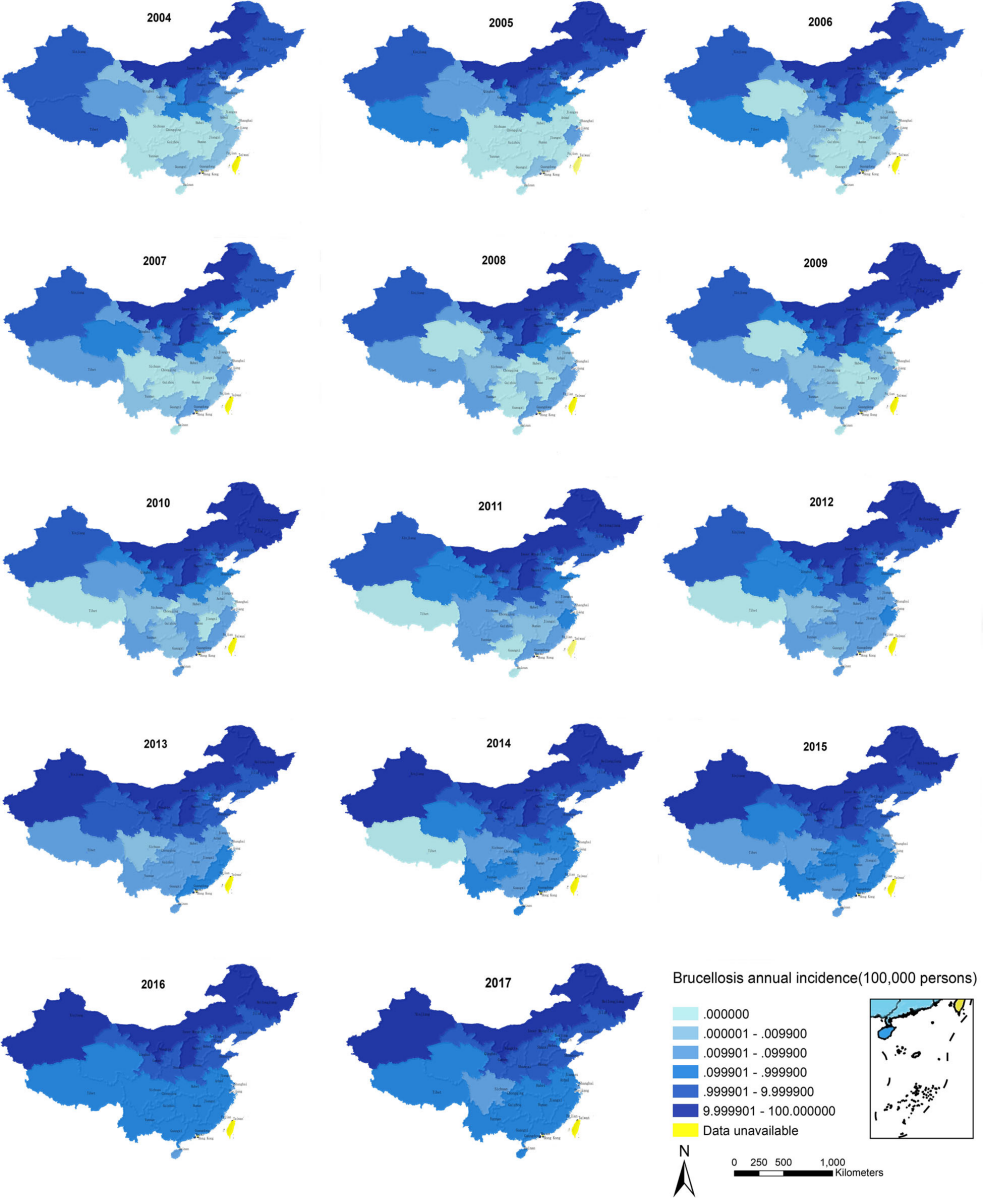
Spatial distribution of brucellosis incidence between 2004 and 2017 in mainland China. Since 2013, brucellosis has occurred throughout the country, with an increasing incidence. The incidence in the northern provinces was higher than that in the southern provinces between 2004 and 2017

**Fig. 4:**
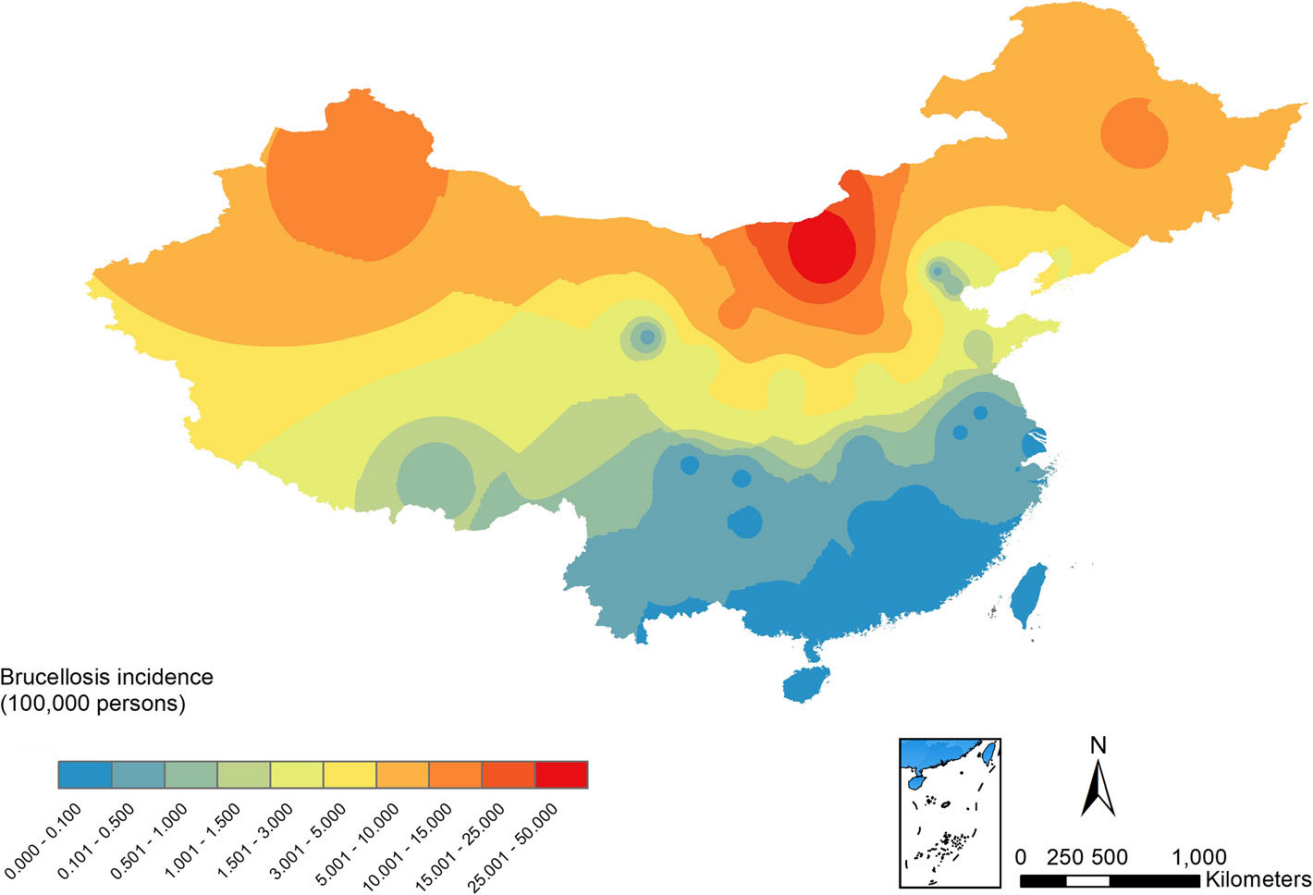
IDW interpolation of average brucellosis incidence between 2004 and 2017 in mainland China. Inverse distance weighting (IDW) estimates the unknown values of the unmeasured points using the weighted average method, which calculates the weight of the unknown points based on their distances to known points. The closer the unknown points are to the known points, the more influence the known will have on the unknown points. The average brucellosis incidence decreased from the north to south between 2004 and 2017. Inner Mongolia was most affected by brucellosis

From 2004 to 2018, there were 530,048 reported cases of human brucellosis in mainland China. Overall, human brucellosis incidence exhibited an increasing trend from 2004 (11,472 cases, 0.88/100,000 persons) and peaked in 2014, with 57,222 cases (4.22/100,000 persons) reported. After that, it decreased slightly, and 40,328 cases were reported in 2018. Most of the brucellosis cases were reported during the period from March to August and concentrated in the period from April to July (Fig. 2). From 2004 to 2017, the years 2004, 2010, and 2016 were selected to compare the quarterly incidence. The second quarter in each year was found to have the highest incidence, and the lowest incidence was found in the fourth quarter (Additional file 1: Figure S1). Other than Tibet, which showed a decreasing trend, human brucellosis incidence in the remaining 30 provinces in China increased over time. Among the 31 provinces, human brucellosis incidence was the highest in the Inner Mongolia Autonomous Region until 2014. The highest incidence was found in Ningxia (43.65/100,000 persons) in 2015 and in Xinjiang (36.56/100,000 persons) in 2016. In 2004, no cases of human brucellosis were reported in Shanghai, Jiangsu, Jiangxi, Hubei, Hunan, Hainan, Chongqing, Sichuan, Guizhou, and Yunnan. After 8 years, human brucellosis cases were reported in all the abovementioned provinces (Fig. 3). Brucellosis hot spots were found in Xinjiang, Inner Mongolia, Heilongjiang, Jilin, Liaoning, Beijing, Tianjin, Hebei, Shanxi, Shaanxi, Ningxia, Gansu, Henan, and Shandong (Additional file 1: Figure S2). The hot spots were all located in northern China, whereas most of the cold spots were in southern China, except Qinghai and Tibet (Additional file 1: Figure S2). The IDW interpolation results also suggested that brucellosis was an endemic disease in northern areas (Fig. 4). With the steadily expanding and increasing trend of epidemics, brucellosis has become a health threat to people living in the southern region.

### Temporal clusters of human brucellosis

High-risk temporal clusters during 2004 to 2017 were detected from February 2011 to August 2017, which was associated with a 1.91-fold higher risk of contracting human brucellosis than the relative months. High-incidence temporal clusters occurred between February and August of each year; the risk was 1.93- to 3.34-fold higher than that in non-high-incidence temporal clusters (Table 1).

**Table 1:** Temporal scan of the number of brucellosis cases between 2004 and 2017 in mainland China

Year	Time frame	Number of cases	Expected cases	Annual cases (1/100,000)	RR	LLR	*P* value
2004	March 2004 to July 2004	7881	4795.25	1.5	3.06	1688.52	< 0.001
2005	February 2005 to July 2005	14,116	9132.22	2.2	3.34	2838.01	< 0.001
2006	February 2006 to July 2006	14,001	9428.36	2.2	2.84	2286.67	< 0.001
2007	March 2007 to August 2007	14,124	9941.55	2.2	2.48	1836.26	< 0.001
2008	March 2008 to August 2008	20,013	13,959.37	3.0	2.55	2735.15	< 0.001
2009	February 2009 to July 2009	25,807	17,760.81	3.9	2.62	3738.03	< 0.001
2010	March 2010 to July 2010	20,745	14,156.48	3.7	2.21	2595.43	< 0.001
2011	February 2011 to July 2011	26,430	18,918.72	4.0	2.29	3032.48	< 0.001
2012	February 2012 to July 2012	27,075	19,649.54	4.0	2.20	2856.34	< 0.001
2013	February 2013 to July 2013	28,463	21,564.29	4.2	1.93	2223.25	< 0.001
2014	March 2014 to August 2014	38,903	28,846.16	5.7	2.09	3619.29	< 0.001
2015	March 2015 to July 2015	33,492	23,888.54	5.8	1.98	3265.38	< 0.001
2016	March 2016 to August 2016	31,495	23,698.30	4.5	1.99	2631.76	< 0.001
2017	February 2017 to July 2017	24,576	19,118.56	3.6	1.79	1563.96	< 0.001
2004–2017	February 2011 to August 2017	310,508	233,673.11	3.5	1.91	24,486.25	< 0.001

RR (relative risk) compares the incidence inside the cluster to that outside the cluster. LLR (log-likelihood ratio) in spatial or space-temporal scanning for disease clusters represents the significant difference between the values inside the cluster and those outside the cluster. LLR is used to evaluate the spatial concentration of cases inside the cluster. Greater RR and LLR values indicate higher risk and more concentrated brucellosis cases inside the cluster than outside the cluster

**Fig. 5:**
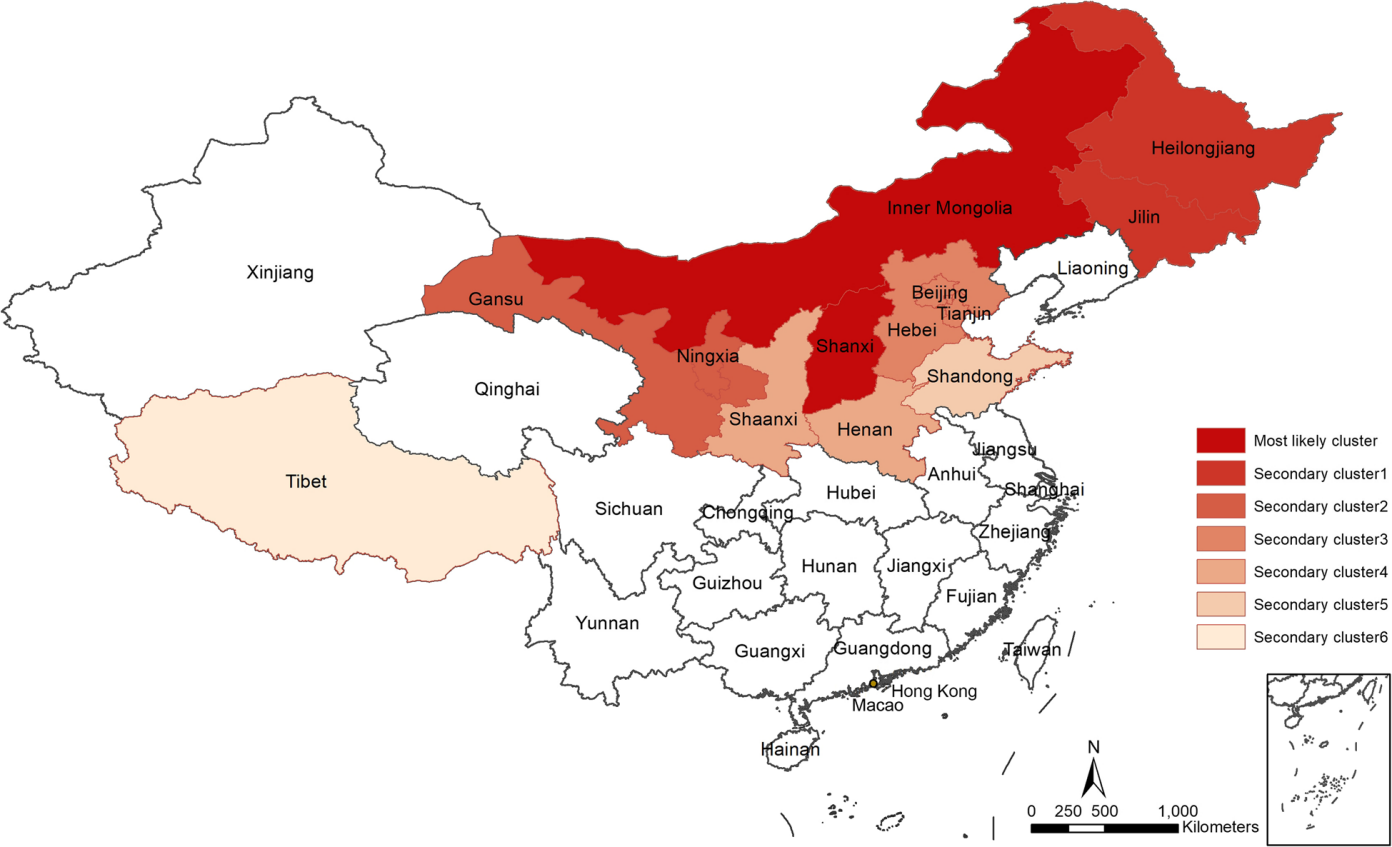
High-incidence spatial clusters between 2004 and 2017 in mainland China. All of the brucellosis clusters were in the northern provinces, and most of the high-incidence spatial clusters were concentrated around Inner Mongolia and Shanxi Province (most likely cluster), except Tibet (secondary cluster 6)

Seven clusters between 2004 and 2017 were identified using a spatial-temporal scan, including one most likely cluster and six secondary clusters (Fig. 5). Inner Mongolia and Shanxi Province accounted for the most likely cluster, with a radius of 369.72 km; 132,946 cases were observed in the most likely cluster. Most of the cases were reported between April 1, 2008, and March 31, 2015. Between 2004 and 2013, Inner Mongolia and Shanxi Province were both identified as most likely clusters, except in 2010 when Inner Mongolia was the only most likely cluster. An expanding trend of the scale of the most likely clusters was observed, and six provinces (Xinjiang, Tibet, Qinghai, Ningxia, Inner Mongolia, and Gansu) had been identified as most likely clusters since 2014 (Additional file 1: Figure S3, Additional file 1: Table S1).

### Socio-economic, environmental, and ecological factors that possibly influence brucellosis incidence

**Fig. 6:**
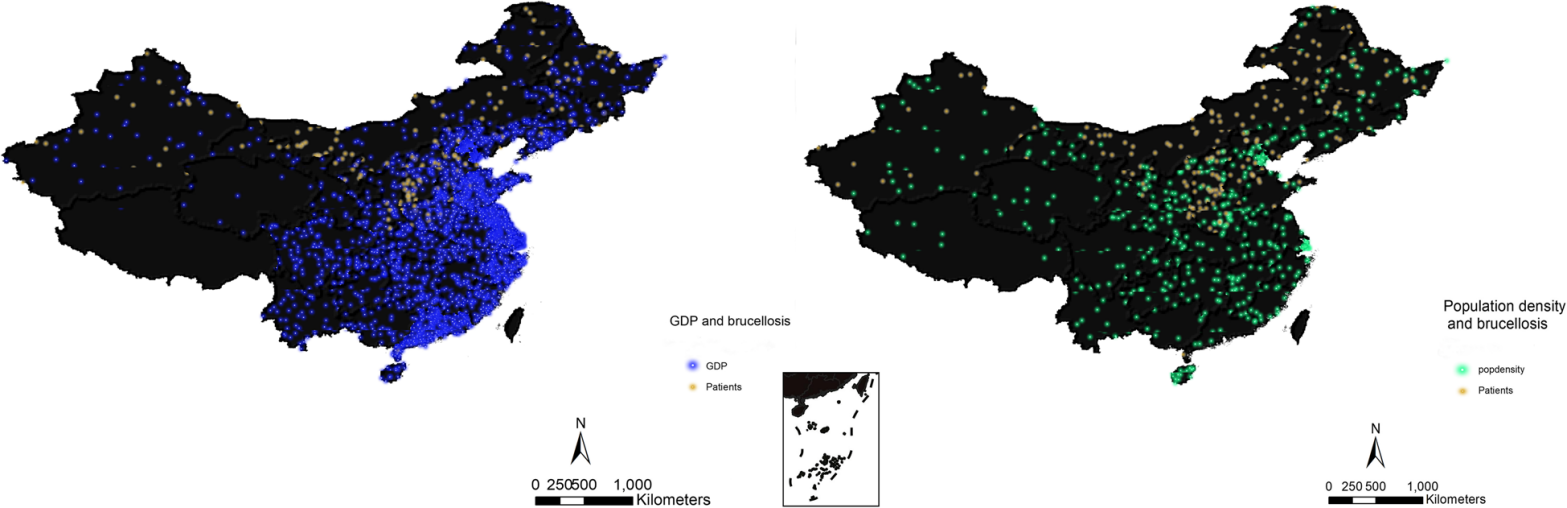
Density map of the relationship between socio-economic factors (GDP and population density) and average brucellosis cases between 2004 and 2017 in mainland China. In both figures, one dot = 200 human brucellosis cases. The GDP and population density of the southeastern coastal and central provinces were higher than those of the northwest regions. Most of the brucellosis cases were distributed in the northern, northwestern, and northeastern provinces

**Fig. 7:**
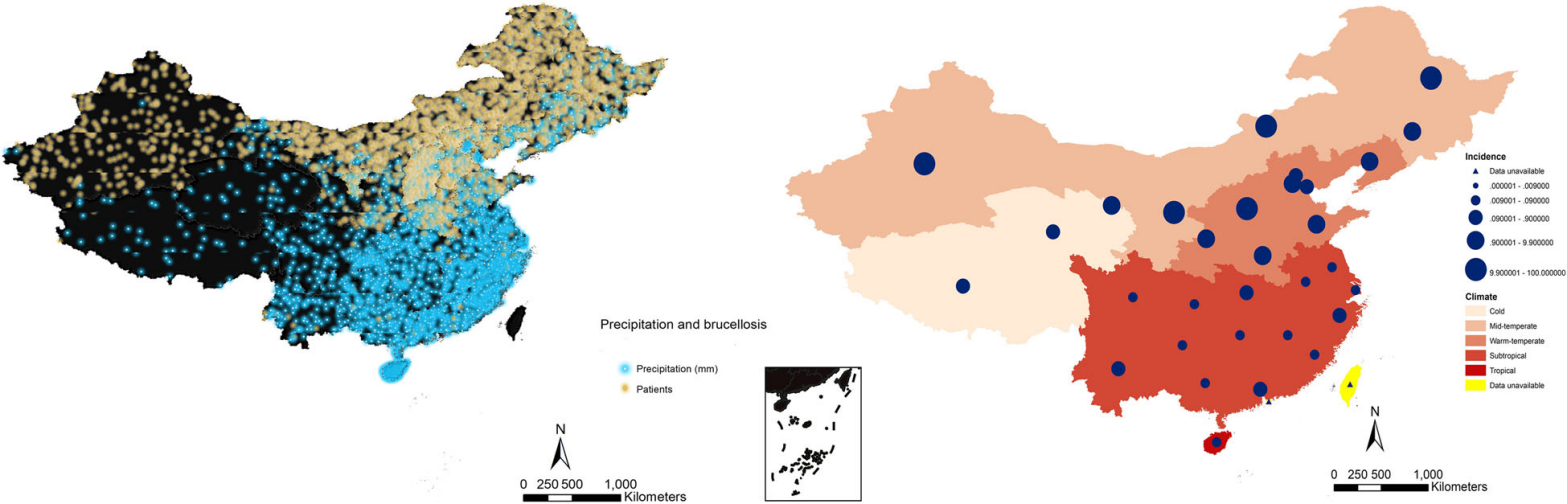
Density map of the relationship between environmental factors (precipitation and climate) and average brucellosis cases between 2004 and 2017 in mainland China. In the left figure, one dot = 10. The southern provinces were considered subtropical and tropical regions and had more precipitation than the warm-temperature, mid-temperate, and cold regions located in northern China

**Fig. 8:**
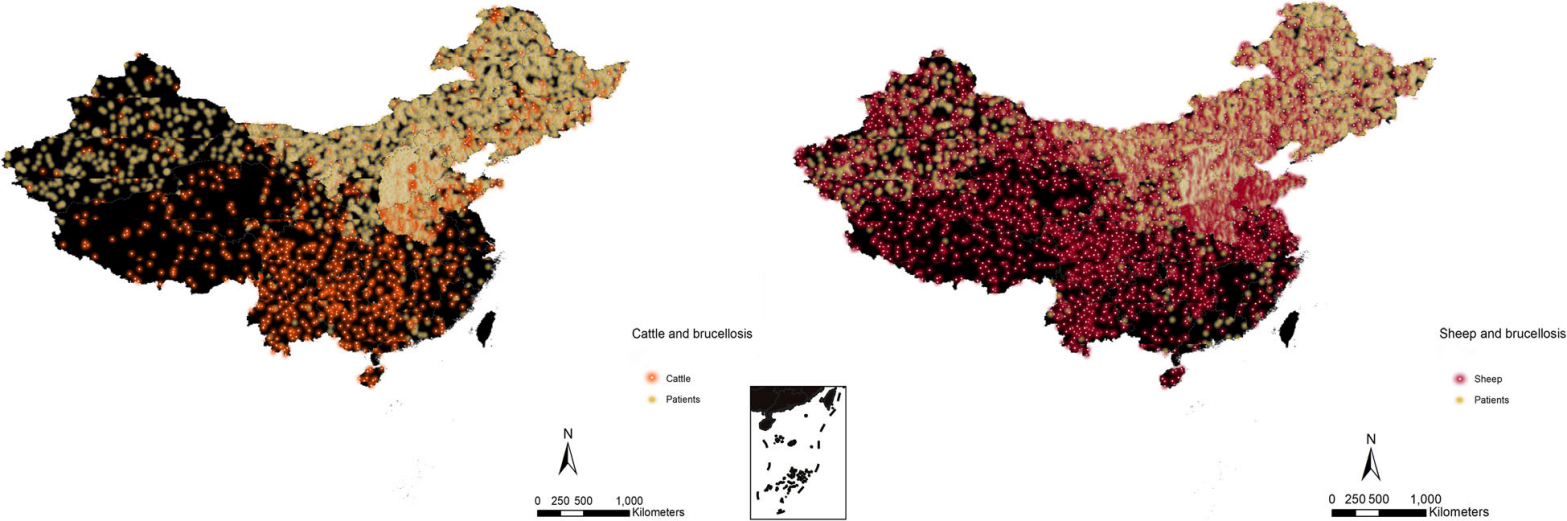
Density map of the relationship between ecological factors (the number of cattle and the number of sheep) and average brucellosis cases between 2004 and 2017 in mainland China. In both figures, one dot = 10. Cattle stocks were evenly distributed in China; however, sheep stocks (caprine and ovine) were concentrated in the north, which coincided with the reported brucellosis cases

The GDP in southeast coastal regions is higher than that in inland regions (Fig. 6). Between 2004 and 2017, Guangdong ($7533), Jiangsu ($6931), and Shandong ($6389) provinces were the top three provinces in terms of GDP (Table 2). Other than Gansu, Guizhou, Hainan, Ningxia, Qinghai, Shanxi, Tibet, and Xinjiang, the GDP in the remaining provinces showed an increasing trend (Additional file 1: Figure S4). The southern provinces with high GDPs had fewer human brucellosis cases between 2004 and 2017 (Fig. 6). The average population densities in Henan (5232.25/km^2^), Shaanxi (4874.18/km^2^), and Jiangxi (4367.26/km^2^) provinces were higher than those in the other provinces in China (Additional file 1: Figure S4, Table 2). The northern provinces are generally characterized as having cold, mid-temperate, or warm-temperate climates. The southern provinces have subtropical and tropical climates (Fig. 7). Tropical, subtropical, and warm-temperate regions had more precipitation than the regions with mid-temperate and cold climates (Additional file 1: Figure S5). The tropical-type, subtropical-type, and high-precipitation areas were associated with lower incidence rates than the other three climate types (Fig. 7). Sheep stocks were larger than cattle stocks (Fig. 8, Table 2). Henan Province had the largest number of cattle stocks, and the Inner Mongolia Autonomous Region had the largest average sheep stock (Additional file 1: Figure S6). Large sheep stocks tended to be associated with more human brucellosis cases than large cattle stocks (Fig. 8).

**Table 2:** Average values of human incidence, the number of sheep, the number of cattle, climate, precipitation, GDP, and population density between 2004 and 2017 in 31 provinces in mainland China

Province	Region	Human incidence (10^4^ person)	Number of sheep (10^4^ heads)	Number of cattle (10^4^ heads)	Climate	Precipitation (mm)	Regional GDP (100 million yuan)	Population density (person/km^2^)
Beijing	North	0.36	74.50	20.78	Warm-temperate	537.42	15,736.47	1379.93
Tianjin	North	0.64	47.83	30.28	Warm-temperate	512.67	10,538.59	2681.40
Hebei	North	4.61	1606.24	471.65	Warm-temperate	533.89	21,575.29	2421.98
Shanxi	North	13.41	857.38	115.15	Warm-temperate	446.41	9478.71	3005.83
Inner Mongolia	North	41.46	5367.81	628.11	Mid-temperate	313.28	11,800.35	1021.80
Liaoning	North	3.01	803.31	343.63	Warm-temperate	693.73	18,554.61	1701.83
Jilin	North	6.01	416.68	459.40	Mid-temperate	615.91	9415.47	2005.94
Heilongjiang	North	12.25	904.04	514.61	Mid-temperate	527.58	10,927.88	4166.81
Shanghai	South	0.01	26.27	6.03	Subtropical	1280.75	18,240.53	3325.74
Jiangsu	South	0.05	523.08	37.09	Subtropical	1045.18	46,400.87	1960.44
Zhejiang	South	0.11	131.98	20.99	Subtropical	1586.29	29,895.27	1772.92
Anhui	South	0.05	641.34	184.91	Subtropical	1057.58	14,348.48	2179.65
Fujian	South	0.08	109.56	70.97	Subtropical	1436.89	16,936.13	2205.97
Jiangxi	South	0.04	66.87	286.34	Subtropical	1667.50	10,872.67	4367.26
Shandong	North	1.23	2329.68	575.21	Warm-temperate	669.86	42,768.47	1388.02
Henan	North	2.01	2182.79	995.71	Warm-temperate	835.17	25,199.60	5232.25
Hubei	South	0.13	409.27	336.08	Subtropical	1156.34	18,655.67	1931.24
Hunan	South	0.06	550.63	453.09	Subtropical	1358.20	18,418.45	3009.53
Guangdong	South	0.12	42.09	246.80	Subtropical	1881.47	50,429.62	2622.58
Guangxi	South	0.09	202.76	470.57	Subtropical	1562.50	10,775.71	1488.38
Hainan	South	0.04	69.99	91.23	Tropical	1710.69	2396.79	2503.08
Chongqing	South	0.04	199.62	128.93	Subtropical	1246.26	9763.17	1679.12
Sichuan	South	0.03	1673.33	986.51	Subtropical	925.99	19,767.96	2376.70
Guizhou	South	0.08	309.02	542.77	Subtropical	1068.51	6193.58	2663.23
Yunnan	South	0.15	938.64	754.04	Subtropical	1159.22	8787.48	2861.49
Tibet	North	0.73	1585.09	617.56	Cold	448.51	641.22	1425.52
Shaanxi	North	2.08	714.99	176.65	Warm-temperate	634.73	11,671.41	4874.18
Gansu	North	2.18	1720.44	430.56	Mid-temperate	394.86	4611.97	4214.25
Qinghai	North	0.33	1507.49	448.99	Cold	389.11	1536.38	2390.73
Ningxia	North	12.11	498.60	97.91	Mid-temperate	262.03	1881.34	1375.01
Xinjiang	North	18.87	3702.89	386.40	Mid-temperate	114.62	6212.04	3609.52

**Fig. 9:**
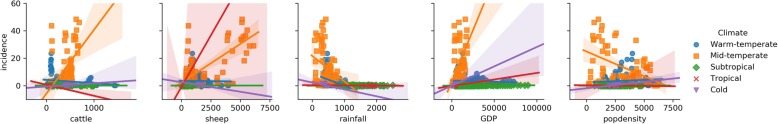
Scatter plots with regression lines representing the association between the number of cattle, the number of sheep, GDP, population density, precipitation, and climate with brucellosis incidence. The blue dots represent those from warm-temperate areas, the yellow squares represent those from mid-temperate areas, the green diamonds represent those from subtropical areas, the red multiplication signs represent those from tropical areas, and the purple upside-down triangles represent those from cold areas. The incidence was higher in the mid-temperate areas than in the other climate areas. In the subtropical areas, the incidence remained at a low level compared with the other climate areas

By Spearman’s correlation analysis, we found that the correlation between rainfall and cattle and sheep stocks was negative, indicating that where rainfall is high, the number of cattle and sheep might be low. Rainfall positively impacted the GDP. The association between subtropical climate and tropical climate and the number of cattle and sheep was negative. Both mid-temperate and warm-temperate climates had a negative correlation with precipitation, and the mid-temperate climate was also negatively correlated with the GDP, suggesting that the GDP might be relatively low in the mid-temperate areas (Table 3). In subtropical and warm-temperate areas, the regression lines showed that the number of cattle, the number of sheep, precipitation, GDP, and population density barely impacted human brucellosis incidence (Fig. 9). In tropical areas, in addition to population density, which did not play a role in the occurrence of brucellosis, the number of cattle negatively influenced human brucellosis incidence; the number of sheep and GDP had a positive correlation with the incidence between 2004 and 2017 (Fig. 9). Cattle stocks were not associated with the brucellosis incidence in cold areas, but sheep stocks, precipitation, GDP, and population density were associated. Human brucellosis incidence in the mid-temperate areas was higher than that in the areas with the other climate types. Among the selected factors, the number of cattle, the number of sheep, and GDP were positively correlated with human brucellosis incidence, and precipitation and population density were negatively correlated in the mid-temperate areas (Fig. 9).

**Table 3:** Spearman’s correlation coefficients between the incidence, cattle stocks, sheep stocks, population density, GDP, precipitation, and climate

	Incidence (10^4^ person)	Cattle (10^4^ heads)	Sheep (10^4^ heads)	Population density (person/km^2^)	GDP (100 million yuan)	Precipitation (mm)	Climate (mid-temperate)	Climate (subtropical)	Climate (warm-temperate)	Climate (tropical)
Incidence (10^4^ person)	1.00	0.22	0.49	0.05	− 0.06	− 0.63	0.52	− 0.72	0.34	− 0.11
Cattle (10^4^ heads)	0.22	1.00	0.71	0.12	− 0.12	− 0.19	0.29	− 0.16	− 0.08	− 0.14
Sheep (10^4^ heads)	0.49	0.71	1.00	0.09	− 0.15	− 0.58	0.39	− 0.47	0.15	− 0.18
Population density (person/km^2^)	0.05	0.12	0.09	1.00	0.01	0.03	− 0.01	− 0.0002	0.008	− 0.05
GDP (100 million yuan)	− 0.06	− 0.12	− 0.15	0.01	1.00	0.48	− 0.42	0.37	0.14	− 0.19
Precipitation (mm)	− 0.63	− 0.19	− 0.58	0.03	0.48	1.00	− 0.59	0.79	− 0.30	0.21
Climate (mid-temperate)	0.52	0.29	0.39	− 0.01	− 0.42	− 0.59	1.00	− 0.45	− 0.36	− 0.07
Climate (subtropical)	− 0.72	− 0.16	− 0.46	− 0.0002	0.32	0.79	− 0.45	1.00	− 0.57	− 0.12
Climate (warm-temperate)	0.34	0.08	0.14	0.10	0.14	− 0.30	− 0.36	− 0.57	1.00	− 0.09
Climate (tropical)	− 0.11	− 0.14	− 0.18	− 0.05	− 0.19	0.21	− 0.07	− 0.12	− 0.09	1.00

“Climate” was a categorical variable and thus encoded using dummy encoding. With dummy encoding, *n* categories will only generate *n* − 1 coded variables. In this case, the climate categorized as cold was dropped and not encoded and was set as the reference climate

The results from the multivariate linear regression analysis showed that the number of sheep, the GDP, a mid-temperate climate, and a warm-temperate climate were significant independent factors of brucellosis incidence in China between 2004 and 2017 (Table 4). The GDP, the sheep stocks, a mid-temperate climate, and a warm-temperate climate were positively associated with human brucellosis incidence. The value of adjusted *R*^2^ was 0.637, meaning that these factors explained 63.7% of the emergence of human brucellosis in China from 2004 to 2017 according to the multivariate linear regression model. The results of the validation of the model (multicollinearity, P-P plot, residual error, linearity check) are compiled in the Additional file (Additional file 1: Table S2, Table S3, and Figure S7).

**Table 4:** Relationship between socio-economic, environmental, ecological factors and brucellosis incidence in mainland China between 2004 and 2017

Risk factors	Coefficient	Standardized coefficient
GDP (log-transformed)^a^	0.7605**	0.2567**
Precipitation (mm)	− 0.0003	− 0.0666
Cattle (10^4^ heads)	− 0.0007	− 0.0734
Sheep (10^4^ heads)	0.0003*	0.1483*
Population density (person/km^2^) (log-transformed)^1^	0.2531	0.0541
Climate (tropical)	1.0761	0.0417
Climate (mid-temperate)	4.9991**	0.7560**
Climate (warm-temperate)	3.1928**	0.5306**

^a^The visualization of GDPs and population density through 2004 and 2017 showed that the distributions of GDPs and population density were right-skewed. Thus, these two factors were log-transformed to normalize the distribution

*P < 0.05

**P < 0.01

## Discussion

On the basis of the monthly number of nationwide human brucellosis cases during 2004–2018 and the monthly and yearly disease data of each province in mainland China from 2004 to 2017, the present study explored the changing distribution of human brucellosis incidence spatially and temporally and investigated the association between the incidence and certain socio-economic, environmental, and ecological factors. Human brucellosis incidence exhibited an obvious seasonal pattern, gradually increasing in January and peaking in May, after which it gradually decreased. The peak incidence occurred in early spring to early summer, and the lowest incidence usually occurred in winter. This result is similar to the results from other studies in China [15, 16] and other countries, such as Italy [17–19]. The identification of seasonal patterns is critical to the success of control and prevention strategies [20]. Sheep breed once or twice a year, mostly in early April and late September; for cattle, the breeding period is from May to July [21]. Since *Brucella* exists in tissues and body fluids in the placenta, fetal membranes, amniotic fluid, breasts, and lymph nodes [2], during the breeding seasons, the risk of exposure to *Brucella* increases correspondingly. In the past, brucellosis cases were concentrated in the northern regions of China, and few southern provinces reported any cases [7]. However, since 2014, all provinces have reported brucellosis cases. In the high-incidence cluster scan analysis, we found that the high-incidence clusters expanded from two provinces (Inner Mongolia and Shanxi) in 2004 to six provinces (Xinjiang, Tibet, Qinghai, Ningxia, Inner Mongolia, and Gansu) in 2017 (Additional file 1: Table S1).

With the help of a multivariate linear model, the contributing factors that potentially drove the emergence and spatial expansion of brucellosis in China were explored. Similarly, as previous studies indicated [15, 22], an increased number of sheep were responsible for an increased number of human brucellosis incidence, and our results supported that conclusion. In addition to linear regression, we conducted ridge regression and Poisson regression to compare the values of the coefficients with those calculated by linear regression and verify the relationship between the potential drivers and human brucellosis incidence in China. The results were similar (Additional file 1: Table S4 and Table S5). Thus, we concluded that the number of sheep was positively associated with the increase in human brucellosis incidence. Our results also revealed that cattle’s involvement in human brucellosis incidence was not significant. In China, *B. melitensis* is the predominant strain. Large-scale serological testing of cattle and sheep found that the positive rate of sheep sera was higher than that of cattle sera [23]. In addition, in high-incidence areas, sheep were the predominant livestock. To cater to the domestic mass demand for meat, the stock of sheep has increased by six times [24]. As sheep are a major influencing factor in the spread of brucellosis, surveillance and corresponding prevention and control measures for sheep populations should be a priority. It has been indicated that changing socio-economic conditions, such as increasing population densities in urban areas and increasing GDPs, may be correlated with the possibility of infection emergence and transmission [13]. Countries with relatively high GDP are normally brucellosis-free [25]. Densely populated areas and areas with poor sanitation and hygiene contribute to the spread of food-borne and soil-borne diseases [26]. With the rapid acceleration of urbanization in developing countries, rural infectious diseases have emerged, posing a significant threat to urban residents [26]. According to the China Statistical Yearbook, the population density in urban areas of China presented an overall increasing trend. In our multivariate linear regression model, no statistically significant correlations between population density and human brucellosis incidence between 2004 and 2017 were found. Urbanization in China does not affect human brucellosis incidence. The Spearman coefficient between the GDP and incidence was − 0.06; however, the GDP was significantly positively correlated with brucellosis incidence in the multivariate analysis. In China, brucellosis cases concentrated in provinces defined as northern areas that have generally lower GDPs and were also categorized in our research as mid-temperate or warm-temperate areas. This positive association was more likely to exist in those provinces that were economically less advantaged in China between 2004 and 2017 than in provinces with high economic advantages. Further analysis should focus on the conditions in which the GDP is positively associated with human brucellosis incidence. We found that among the five climate zones, the mid-temperate climate and warm-temperate climate significantly influenced the emergence of brucellosis in China, with cold climate as the reference climate. There have been some hypotheses regarding rainfall and climate changes as environmental drivers for vector-borne diseases [27, 28]. The present study did not find such a pattern among precipitation and human brucellosis events. This could be because in the model, the variable precipitation was not enough to explain the whole picture, unlike climate [11]. Thus, further study on brucellosis epidemiology regarding ecologic factors should focus on the characteristics and differences in and between each climate type to identify the determinants.

Globally, zoonoses account for 60.3% of EIDs [11], and livestock account for up to 37% of the agricultural GDP [29]. Over the past 14 years, China’s economy has entered a high-speed growth phase, with the per capita GDP increasing from 1828 US dollars in 2004 to 8828 US dollars in 2017 [30]. The increase in the demand for high living standards stimulates the demand for meat, which consequently causes an increase in livestock farming and meat importation [31]; thus, the risk of exposure to animals, either live or slaughtered, is increasing. In China, the amount of food and animal imports is approximately 7–8 times higher than the amount of exports, and importation is increasing at a rate of 15% per year [31]. International trade could facilitate the spread and introduction of new types of *Brucella* pathogen from other countries, which might increase the difficulty of control and prevention. In light of the expanding scale and increasing incidence of brucellosis in China, we found that the human brucellosis incidence increased more rapidly in certain provinces, such as Heilongjiang, Shandong, Henan, Gansu, Ningxia, Zhejiang, and Guangdong provinces. The driving and influencing factors of the spread should be elucidated. Our results suggested that direct contact with the infectious livestock remains the primary source of human brucellosis. The reasons behind the increasing incidence in the southern provinces in China could be travel and the increasing demand for mutton and beef, which has led to an increase in the transportation of cattle, sheep, and other animal products from the northern pastoral areas, such as Inner Mongolia [32]. It is critical to strengthen the management and supervision of the agricultural market and the international and domestic transportation of livestock to prevent contaminated meat and dairy products from entering the market [3].

Occupational groups, such as slaughterhouse workers, meat-packing employees, veterinarians, and herdsmen, still account for the majority of brucellosis patients. A study in Pakistan showed that inadequate understanding of brucellosis can lead to risky behaviors that could cause infection [10, 33]. With the development of economic globalization, the amount of air travel passengers increased by five times from 2004 to 2017 [34]. There have been several reports about brucellosis infection among returning travelers [35, 36]. Health education for travelers should also be a priority, especially for travelers visiting high-risk regions during high-risk periods. The focus of health education should be avoiding the consumption of undercooked meat and unpasteurized dairy products and maintaining a safe distance from livestock. For medical practitioners, especially those who work in non-brucellosis-endemic areas, if patients complain of fever, sweating, joint pain, and malaise, they should be asked about their travel history and exposure history to avoid a misdiagnosis or late diagnosis of human brucellosis.

“One Health” has been defined as a collaborative, multifaceted, and multidisciplinary approach to address local, regional, national, and worldwide health problems. A strength of the present study was that it was conducted at the livestock-human-ecological interface. We mapped the changing epidemiology of brucellosis between 2004 and 2017 in 31 provinces of China, detected “hot spot” areas and time frames, and quantified the impact of representative socio-economic, environmental, and ecological factors on brucellosis incidence.

There are some limitations to our study. First, data regarding human brucellosis incidence were collected by passive monitoring due to the national policy. Most brucellosis cases occurred in farmers, who often do not have adequate access to a timely diagnosis and effective treatment. Thus, the brucellosis incidence identified in this analysis might underestimate the real extent of the brucellosis situation in China. Second, we macroscopically described the changing trend of human brucellosis incidence throughout the whole country using province as the study unit. Thus, the association between the changing incidence and the potentially driving factors might not accurately describe the local relationship. Future studies should divide the whole country into several categories based on socio-economic or ecological characteristics, such as geographical locations, climate conditions, vegetation coverage, vegetation type, people’s living standards, prices of meat, or meat consumption. In addition, other machine learning models can be used to analyze the changing epidemiology of human brucellosis, explore the influencing factors and their interactions, examine their associations, and further predict the future trends of human brucellosis incidence under these classifications.

## Conclusions

In summary, the geographical extent of human brucellosis in China has been expanding with an increasing incidence in each province. The human brucellosis incidence and the high-incidence clusters were concentrated in the northern areas but were also observed to be expanding. Most cases were reported during the early spring to early summer (February–August). The inventory of sheep, GDP, and climate type were significantly associated with the incidence of human brucellosis in China between 2004 and 2017.

## Supplementary information

**Additional file 1: Table** **S1.** Spatio-temporal scan analysis of high-incidence clusters between 2004 and 2017 in mainland China. **Table S2.** VIFs of the number of cattle, the number of sheep, GDP, population density, rainfall and climate in the multivariate linear regression model. **Table S3.** VIF of the number of cattle, the number of sheep, GDP, population density, rainfall, Tropical climate, Mid-temperate climate and Warm-temperate climate in the multivariate linear regression model. **Table S4.** Coefficients of the number of sheep, GDP, population density, rainfall and climate in the ridge regression model. **Table S5.** Coefficients of the number of sheep, GDP, population density, rainfall and climate in the Poisson regression model. **Figure S1.** Spatial distribution of quarterly brucellosis incidence (100,000 persons) in 2004, 2010 and 2016 in mainland China. **Figure S2.** Hot and cold spots of brucellosis incidence between 2004 and 2017 in mainland China. **Figure S3.** Spatio-temporal distribution of high-incidence clusters between 2004 and 2017 in mainland China. **Figure S4.** GDP (left) and population density (right) provincial distribution in mainland China between 2004 and 2017. **Figure S5.** Climate and Precipitation distribution in provinces of mainland China between 2004 and 2017. **Figure S6.** Cattle stocks (left) and Sheep stocks (right) provincial distribution in mainland China between 2004 and 2017. **Figure S7.** Validation of multivariate linear model: (a) P-P plot, (b) Residual error and (c) linearity check.

## Data Availability

The datasets analyzed during the current study are available from the corresponding author on reasonable request.

## Abbreviations

CMDC: China Meteorological Data Service Center
EIDs: Emerging infectious diseases
GDP: Gross domestic product
IDW: Inversed distance weighted
LLR: Log-likelihood ratio
NIDR: National Noticeable Infectious Disease Reporting
VIF: Variance inflation factor
