# Supplementary material for: Spatial-temporal distribution of human brucellosis in mainland China from 2004 to 2017 and an analysis of social and environmental factors
Source: Environ Health Prev Med. 2020 Jan 2;25:1. doi: 10.1186/s12199-019-0839-z (PMC6941396; doi:10.1186/s12199-019-0839-z)
Supplement: Supplementary file 1 — Additional file 1: Table S1. Spatio-temporal scan analysis of high-incidence clusters between 2004 and 2017 in mainland China. Table S2. VIFs of the number of cattle, the number of sheep, GDP, population density, rainfall and climate in the multivariate linear regression model. Table S3. VIF of the number of cattle, the number of sheep, GDP, population density, rainfall, Tropical climate, Mid-temperate climate and Warm-temperate climate in the multivariate linear regression model. Table S4. Coefficients of the number of sheep, GDP, population density, rainfall and climate in the ridge regression model. Table S5. Coefficients of the number of sheep, GDP, population density, rainfall and climate in the Poisson regression model. Figure S1. Spatial distribution of quarterly brucellosis incidence (100,000 persons) in 2004, 2010 and 2016 in mainland China. Figure S2. Hot and cold spots of brucellosis incidence between 2004 and 2017 in mainland China. Figure S3. Spatio-temporal distribution of high-incidence clusters between 2004 and 2017 in mainland China. Figure S4. GDP (left) and population density (right) provincial distribution in mainland China between 2004 and 2017. Figure S5. Climate and Precipitation distribution in provinces of mainland China between 2004 and 2017. Figure S6. Cattle stocks (left) and Sheep stocks (right) provincial distribution in mainland China between 2004 and 2017. Figure S7. Validation of multivariate linear model: (a) P-P plot, (b) Residual error and (c) linearity check. [file 12199_2019_839_MOESM1_ESM.docx]

Spatial-temporal distribution of human brucellosis in mainland China from 2004 to 2017 and an analysis of social and environmental factors

Cheng Peng^1^, Yan-Jun Li^1^, De-Sheng Huang^1,2^, Peng Guan*

**Supplementary Material**

**Table S1 Spatio-temporal scan analysis of high-incidence clusters between 2004 and 2017 in mainland China**

| **Year** | **Spatial cluster** | **Locations included** | **Time frame** | **Number of cases** | **Expected cases** | **RR** | **LLR** | ***P*-value** |
| --- | --- | --- | --- | --- | --- | --- | --- | --- |
| **2004** | Most likely cluster | Inner Mongolia, Shanxi | 2004/3/1 to 2004/8/31 | 4544 | 256.52 | 28.68 | 9723.86 | 0.001 |
|  | Secondary likely cluster1 | Liaoning, Jilin, Heilongjiang | 2004/2/1 to 2004/7/31 | 2878 | 475.87 | 7.74 | 3061.33 | 0.001 |
|  | Secondary likely cluster2 | Tibet | 2004/2/1 to 2004/6/30 | 206 | 1.01 | 206.79 | 891.47 | 0.001 |
|  | Secondary likely cluster3 | Beijing, Tianjin, Hebei | 2004/4/1 to 2004/6/30 | 336 | 206.55 | 1.65 | 34.78 | 0.001 |
|  | Secondary likely cluster4 | Shaanxi | 2004/2/1 to 2004/5/31 | 168 | 108.4 | 1.56 | 14.16 | 0.001 |
| **2005** | Most likely cluster | Inner Mongolia, Shanxi | 2005/2/1 to 2005/7/31 | 8521 | 408.88 | 37.93 | 19952.68 | 0.001 |
|  | Secondary likely cluster1 | Heilongjiang | 2005/2/1 to 2005/7/31 | 3009 | 271.26 | 13.06 | 4720.57 | 0.001 |
|  | Secondary likely cluster2 | Liaoning, Jilin | 2005/1/1 to 2005/6/30 | 854 | 492.6 | 1.77 | 112.17 | 0.001 |
|  | Secondary likely cluster3 | Beijing, Tianjin, Hebei | 2005/3/1 to 2005/6/30 | 660 | 451.45 | 1.48 | 43.32 | 0.001 |
|  | Secondary likely cluster4 | Shaanxi | 2005/3/1 to 2005/8/31 | 358 | 266.37 | 1.35 | 14.44 | 0.001 |
| **2006** | Most likely cluster | Inner Mongolia, Shanxi | 2006/2/1 to 2006/7/31 | 8387 | 421.47 | 34.82 | 19138.68 | 0.001 |
|  | Secondary likely cluster1 | Heilongjiang | 2006/2/1 to 2006/7/31 | 2283 | 278.29 | 9.19 | 2911.37 | 0.001 |
|  | Secondary likely cluster2 | Beijing, Tianjin, Hebei | 2006/1/1 to 2006/6/30 | 1685 | 696.92 | 2.56 | 526.67 | 0.001 |
|  | Secondary likely cluster3 | Shaanxi | 2006/4/1 to 2006/7/31 | 386 | 181.49 | 2.15 | 87.89 | 0.001 |
|  | Secondary likely cluster4 | Liaoning, Jilin | 2006/3/1 to 2006/7/31 | 654 | 430.36 | 1.54 | 51.40 | 0.001 |
| **2007** | Most likely cluster | Inner Mongolia, Shanxi | 2007/3/1 to 2007/8/31 | 8788 | 443.89 | 34.91 | 20036.76 | 0.001 |
|  | Secondary likely cluster1 | Jilin, Heilongjiang | 2007/2/1 to 2007/7/31 | 2395 | 491.55 | 5.41 | 1986.70 | 0.001 |
|  | Secondary likely cluster2 | Beijing, Tianjin, Hebei | 2007/2/1 to 2007/7/31 | 1665 | 730.05 | 2.40 | 461.19 | 0.001 |
|  | Secondary likely cluster3 | Shaanxi | 2007/3/1 to 2007/7/31 | 492 | 235.08 | 2.12 | 108.15 | 0.001 |
| **2008** | Most likely cluster | Inner Mongolia, Shanxi | 2008/3/1 to 2008/8/31 | 11464 | 621.85 | 30.7 | 25097.12 | 0.001 |
|  | Secondary likely cluster1 | Jilin, Heilongjiang | 2008/2/1 to 2008/7/31 | 4333 | 689.05 | 7.27 | 4580.15 | 0.001 |
|  | Secondary likely cluster2 | Beijing, Tianjin, Hebei | 2008/3/1 to 2008/7/31 | 2056 | 877.49 | 2.45 | 598.28 | 0.001 |
|  | Secondary likely cluster3 | Shaanxi | 2008/4/1 to 2008/7/31 | 581 | 261.82 | 2.25 | 145.79 | 0.001 |
| **2009** | Most likely cluster | Inner Mongolia, Shanxi | 2009/3/1 to 2009/8/31 | 15328 | 802.27 | 32.65 | 34237.96 | 0.001 |
|  | Secondary likely cluster1 | Jilin, Heilongjiang | 2009/2/1 to 2009/7/31 | 6062 | 880.51 | 8.08 | 6918.68 | 0.001 |
|  | Secondary likely cluster2 | Beijing, Tianjin, Hebei | 2009/2/1 to 2009/7/31 | 2351 | 1357.38 | 1.78 | 312.22 | 0.001 |
|  | Secondary likely cluster3 | Shaanxi | 2009/3/1 to 2009/6/30 | 519 | 336.88 | 1.55 | 42.65 | 0.001 |
| **2010** | Most likely cluster | Inner Mongolia | 2010/3/1 to 2010/8/31 | 10959 | 315.52 | 50.94 | 30143.83 | 0.001 |
|  | Secondary likely cluster1 | Jilin, Heilongjiang | 2010/2/1 to 2010/7/31 | 5657 | 826.15 | 8.02 | 6425.35 | 0.001 |
|  | Secondary likely cluster2 | Shanxi | 2010/2/1 to 2010/7/31 | 2748 | 448.74 | 6.58 | 2761.86 | 0.001 |
|  | Secondary likely cluster3 | Beijing, Tianjin, Hebei | 2010/3/1 to 2010/7/31 | 1579 | 1109.62 | 1.44 | 91.04 | 0.001 |
| **2011** | Most likely cluster | Inner Mongolia, Shanxi | 2011/2/1 to 2011/7/31 | 15763 | 857.43 | 30.62 | 34469.23 | 0.001 |
|  | Secondary likely cluster1 | Jilin, Heilongjiang | 2011/1/1 to 2011/6/30 | 5068 | 929.13 | 6.14 | 4697.92 | 0.001 |
|  | Secondary likely cluster2 | Beijing, Tianjin, Hebei | 2011/4/1 to 2011/6/30 | 1312 | 753.24 | 1.77 | 173.50 | 0.001 |
| **2012** | Most likely cluster | Inner Mongolia, Shanxi | 2012/2/1 to 2012/7/31 | 12682 | 889.4 | 20.53 | 23926.20 | 0.001 |
|  | Secondary likely cluster1 | Heilongjiang | 2012/2/1 to 2012/7/31 | 5355 | 558.92 | 10.93 | 7613.19 | 0.001 |
|  | Secondary likely cluster2 | Liaoning, Jilin | 2012/3/1 to 2012/8/31 | 2296 | 1052.16 | 2.26 | 568.12 | 0.001 |
|  | Secondary likely cluster3 | Beijing, Tianjin, Hebei | 2012/2/1 to 2012/7/31 | 2721 | 1570.05 | 1.79 | 362.94 | 0.001 |
| **2013** | Most likely cluster | Inner Mongolia, Shanxi | 2013/2/1 to 2013/7/31 | 10161 | 975.13 | 13.29 | 15701.70 | 0.001 |
|  | Secondary likely cluster1 | Heilongjiang | 2013/2/1 to 2013/7/31 | 5057 | 610.25 | 9.25 | 6486.07 | 0.001 |
|  | Secondary likely cluster2 | Liaoning, Jilin | 2013/2/1 to 2013/7/31 | 2692 | 1136.33 | 2.46 | 795.06 | 0.001 |
|  | Secondary likely cluster3 | Beijing, Tianjin, Hebei | 2013/2/1 to 2013/7/31 | 3493 | 1737.67 | 2.10 | 720.97 | 0.001 |
|  | Secondary likely cluster4 | Gansu, Ningxia | 2013/4/1 to 2013/8/31 | 853 | 435.28 | 1.98 | 158.19 | 0.001 |
|  | Secondary likely cluster5 | Henan, Shaanxi | 2013/4/1 to 2013/7/31 | 1973 | 1413.33 | 1.41 | 102.28 | 0.001 |
| **2014** | Most likely cluster | Inner Mongolia, Tibet, Gansu, Qinghai, Ningxia, Xinjiang | 2014/3/1 to 2014/8/31 | 14621 | 1896.39 | 10.01 | 18729.40 | 0.001 |
|  | Secondary likely cluster1 | Hebei, Shanxi | 2014/2/1 to 2014/7/31 | 10171 | 2297.63 | 5.17 | 7851.06 | 0.001 |
|  | Secondary likely cluster2 | Heilongjiang | 2014/2/1 to 2014/7/31 | 3925 | 798.30 | 5.21 | 3212.70 | 0.001 |
|  | Secondary likely cluster3 | Henan, Shaanxi | 2014/3/1 to 2014/7/31 | 4131 | 2325.81 | 1.84 | 597.89 | 0.001 |
|  | Secondary likely cluster4 | Liaoning, Jillin | 2014/2/1 to 2014/7/31 | 2884 | 1487.67 | 1.99 | 530.43 | 0.001 |
| **2015** | Most likely cluster | Inner Mongolia, Tibet, Gansu, Qinghai, Ningxia, Xinjiang | 2015/3/1 to 2015/8/31 | 14363 | 1896.76 | 9.79 | 18142.53 | 0.001 |
|  | Secondary likely cluster1 | Hebei, Shanxi | 2015/2/1 to 2015/7/31 | 8492 | 2285.97 | 4.19 | 5304.48 | 0.001 |
|  | Secondary likely cluster2 | Liaoning, Jilin, Heilongjiang | 2015/2/1 to 2015/7/31 | 6973 | 2256.69 | 3.38 | 3359.55 | 0.001 |
|  | Secondary likely cluster3 | Henan, Shaanxi | 2015/3/1 to 2015/7/31 | 4136 | 2312.91 | 1.85 | 611.58 | 0.001 |
|  | Secondary likely cluster4 | Shandong | 2015/4/1 to 2015/5/31 | 979 | 684.12 | 1.44 | 56.77 | 0.001 |
| **2016** | Most likely cluster | Inner Mongolia, Tibet, Gansu, Qinghai, Ningxia, Xinjiang | 2016/3/1 to 2016/8/31 | 12275 | 1567.53 | 10.24 | 15925.19 | 0.001 |
|  | Secondary likely cluster1 | Liaoning, Jilin, Heilongjiang | 2016/2/1 to 2016/7/31 | 6195 | 1853.39 | 3.7 | 3349.17 | 0.001 |
|  | Secondary likely cluster2 | Shanxi | 2016/3/1 to 2016/8/31 | 3086 | 632.37 | 5.15 | 2504.05 | 0.001 |
|  | Secondary likely cluster3 | Henan, Shaanxi | 2016/2/1 to 2016/7/31 | 3492 | 2267.05 | 1.58 | 300.45 | 0.001 |
|  | Secondary likely cluster4 | Shandong | 2016/3/1 to 2016/6/30 | 1874 | 1132.72 | 1.68 | 208.20 | 0.001 |
|  | Secondary likely cluster5 | Beijing, Tianjin, Hebei | 2016/2/1 to 2016/6/30 | 2428 | 1579.28 | 1.57 | 203.51 | 0.001 |
| **2017** | Most likely cluster | Inner Mongolia, Tibet, Gansu, Qinghai, Ningxia, Xinjiang | 2017/3/1 to 2017/8/31 | 10678 | 1290.29 | 11.06 | 14474.88 | 0.001 |
|  | Secondary likely cluster1 | Liaoning, Jilin, Heilongjiang | 2017/2/1 to 2017/7/31 | 4758 | 1497.58 | 3.48 | 2387.57 | 0.001 |
|  | Secondary likely cluster2 | Shanxi | 2017/3/1 to 2017/8/31 | 2016 | 518.24 | 4.05 | 1270.71 | 0.001 |
|  | Secondary likely cluster3 | Shandong | 2017/2/1 to 2017/6/30 | 1832 | 1141.91 | 1.63 | 182.31 | 0.001 |
|  | Secondary likely cluster4 | Henan, Shaanxi | 2017/4/1 to 2017/6/30 | 1247 | 927.33 | 1.36 | 51.04 | 0.001 |
|  | Secondary likely cluster5 | Beijing, Tianjin, Hebei | 2017/2/1 to 2017/7/31 | 1921 | 1548.94 | 1.25 | 43.36 | 0.001 |
| **2004-2017** | Most likely cluster | Inner Mongolia, Shanxi | 2008/4/1 to 2015/3/31 | 132946 | 11024.81 | 16.21 | 226236.60 | 0.001 |
|  | Secondary likely cluster1 | Jilin, Heilongjiang | 2009/1/1 to 2015/12/31 | 56697 | 11991.59 | 5.22 | 45547.13 | 0.001 |
|  | Secondary likely cluster2 | Gansu, Ningxia | 2014/3/1 to 2016/11/30 | 15770 | 3344.79 | 4.84 | 12190.41 | 0.001 |
|  | Secondary likely cluster3 | Beijing, Tianjin, Hebei | 2012/1/1 to 2016/7/31 | 25673 | 13138.68 | 2.01 | 4830.73 | 0.001 |
|  | Secondary likely cluster4 | Henan, Shaanxi | 2014/2/1 to 2016/7/31 | 16971 | 8630.78 | 2.00 | 3208.20 | 0.001 |
|  | Secondary likely cluster5 | Shandong | 2015/1/1 to 2017/6/30 | 9694 | 6453.13 | 1.51 | 714.98 | 0.001 |
|  | Secondary likely cluster6 | Tibet | 2004/3/1 to 2004/6/30 | 197 | 2.40 | 82.07 | 673.64 | 0.001 |

**Table S2. VIFs of the number of cattle, the number of sheep, GDP, population density, rainfall and climate in the multivariate linear regression model**

| **Risk factors** | **Coefficient** | **Standardized coefficient** | **VIF** |
| --- | --- | --- | --- |
| GDP (log-transformation) | 1.0607** | 0.5908** | 2.030 |
| Precipitation | 0.0003 | 0.0464 | 4.252 |
| Cattle | -0.0007 | -0.1338 | 1.666 |
| Sheep | 0.0002* | 0.4476* | 2.490 |
| Population density(log-transformation) | 0.1364 | 0.0859 | 1.140 |
| Climate (Tropical) | -3.2059* | -2.7829* | 1.809 |
| Climate (Mid-temperate) | 1.5808* | 1.3722* | 9.050 |
| Climate (Subtropical) | -4.6725** | -4.0561** | 15.479^1^ |
| Climate (Warm-temperate) | -0.7223 | -0.6270 | 12.226^1^ |

Note: ^1^Variance inflation factor (VIF) is the index to examine the multicollinearity. VIF > 10 tells a high multicollinearity in a linear regression model. In our case, Climate (Mid-temperate), Climate (Subtropical) and Climate (Warm-temperate) were dummy variables representing a categorical variable (Climate) with five categories. Thus, the high-VIF situation can be safely dismissed and these variables were still included in the multivariate regression model [1]. Still, we eliminated the factor with highest VIF value trying to improve the performance of the multivariate linear regression model, in this case Subtropical climate factor was removed. The new result of VIFs of each factor is presented in Table S3.

The climate was categorized following the previous study, northern provinces were temperate climate, among which there were Cold, Mid-temperate and Warm-temperate climate; southern provinces were subtropical climate, Hainan province was Tropical climate [2,3].

**Table S3. VIF of the number of cattle, the number of sheep, GDP, population density, rainfall, Tropical climate, Mid-temperate climate and Warm-temperate climate in the multivariate linear regression model**

| **Risk factors** | **Coefficient** | **Standardized coefficient** | **VIF** |
| --- | --- | --- | --- |
| GDP (log-transformation) | 0761** | 0.257** | 1.793 |
| Precipitation | -0.0004 | -0.0665 | 3.953 |
| Cattle | -0.0007 | -0.0734 | 1.666 |
| Sheep | 0.0003* | 0.1483* | 2.458 |
| Population density(log-transformation) | 0.2531 | 0.0541 | 1.125 |
| Climate (Tropical) | 1.0761 | 0.0417 | 1.175 |
| Climate (Mid-temperate) | 4.999* | 0.7560* | 2.893 |
| Climate (Warm-temperate) | 3.1928 | 0.5306 | 2.473 |

**Table S4. Coefficients of the number of sheep, GDP, population density, rainfall and climate in the ridge regression model**

| **Risk factors** | **Coefficient (linear regression)** | **Coefficient (ridge regression)** |
| --- | --- | --- |
| GDP (log-transformed) | 0.7605** | 0.7596** |
| Sheep (10^4^ heads) | 0.0003* | 0.0003* |
| Climate (Mid-temperate) | 4.9992* | 4.9571** |
| Climate (Warm-temperate) | 3.1928** | 3.1635** |

Note: * *P* < 0.05; ** *P* < 0.01

From the previous linear regression, we had five significant associated factors, so in the ridge regression analysis only those five significant factors were chosen.

**Table S5. Coefficients of the number of sheep, GDP, population density, rainfall and climate in the Poisson regression model**

| **Risk factors** | **Coefficient (linear regression)** | **Coefficient (Poisson regression)** |
| --- | --- | --- |
| GDP (log-transformed) | 0.7605** | 0.5275** |
| Sheep (10^4^ heads) | 0.0003* | 0.0002** |
| Climate (Mid-temperate) | 4.9992* | 4.2338** |
| Climate (Warm-temperate) | 3.1928** | 2.8571** |

Note: * *P* < 0.05; ** *P* < 0.01

From the previous linear regression, we had five significant associated factors, so in the Poisson regression analysis only those five significant factors were chosen.


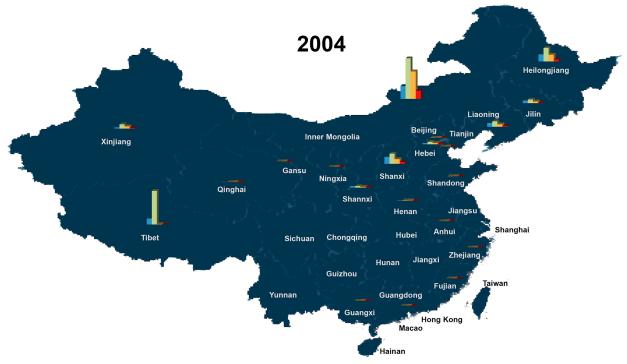

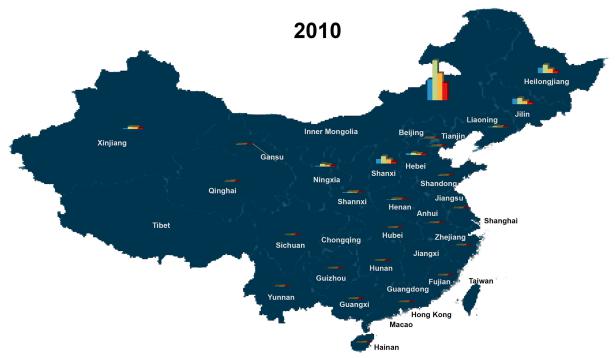

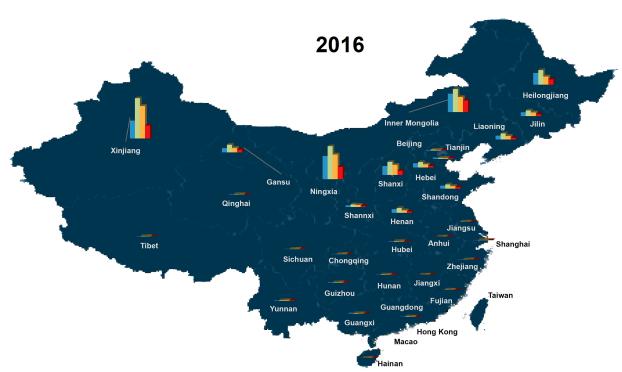

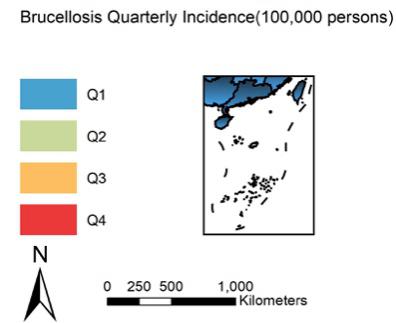


**Figure S1** Spatial distribution of quarterly brucellosis incidence (100,000 persons) in 2004, 2010 and 2016 in mainland China. Overall quarterly incidence was increasing among 31 provinces. Brucellosis incidence in the second quarter (April to June) was the highest among four quarters in 2004, 2010 and 2016.


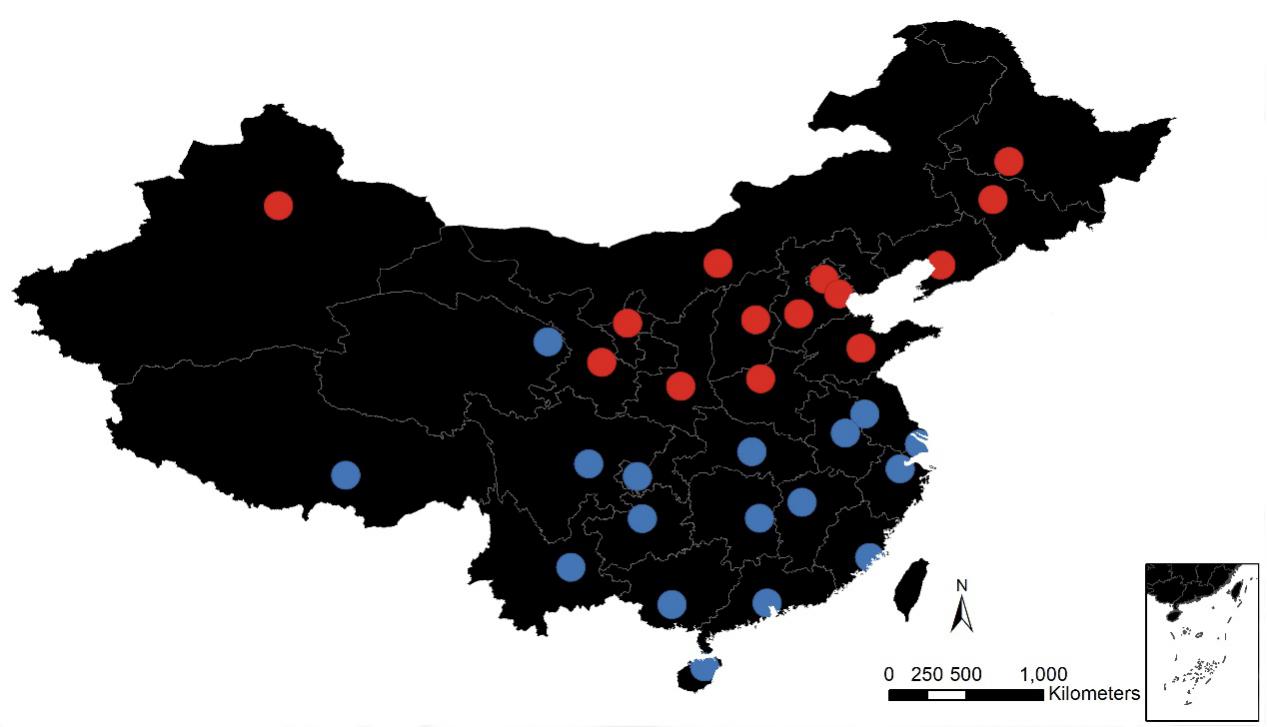


**Figure S2** Hot and cold spots of brucellosis incidence between 2004 and 2017 in mainland China. Red dots represented hot spots with 99% confidence, which distributed in northern China. Blue dots represented cold spots with 99% confidence, which distributed in southern China. (Note: An element with statistically significant high value that detected as hotspot not only has high values, but also get surrounded by other elements with high values to prove that the spatial pattern is not random.)


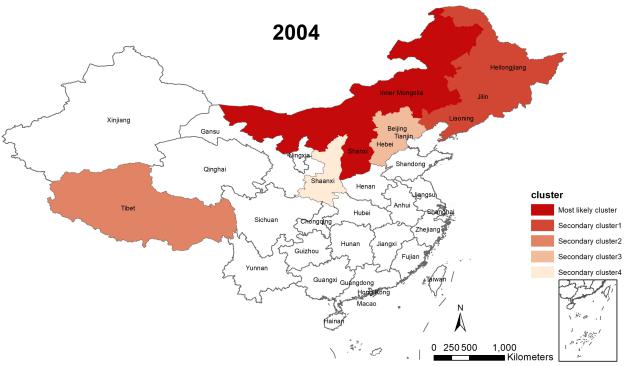

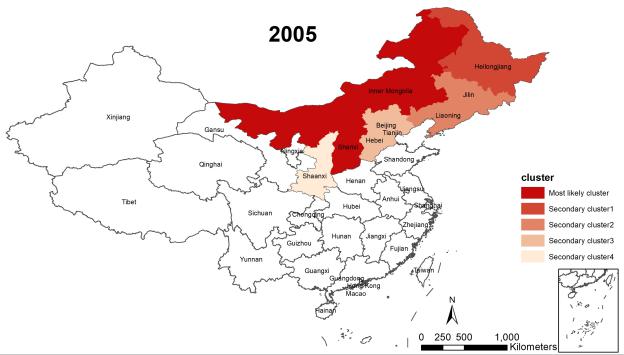

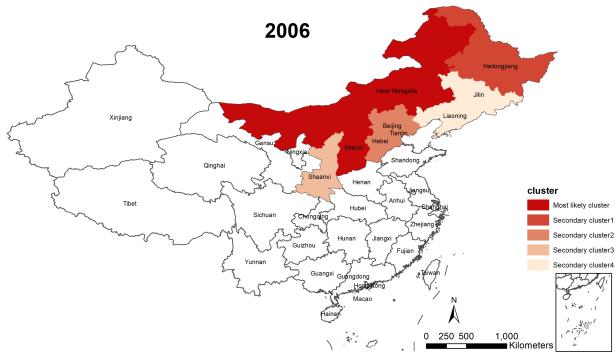

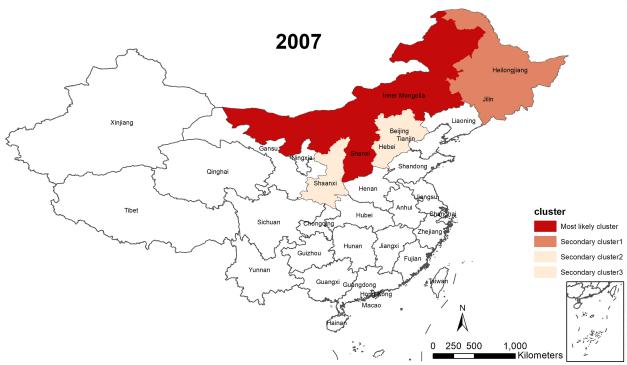

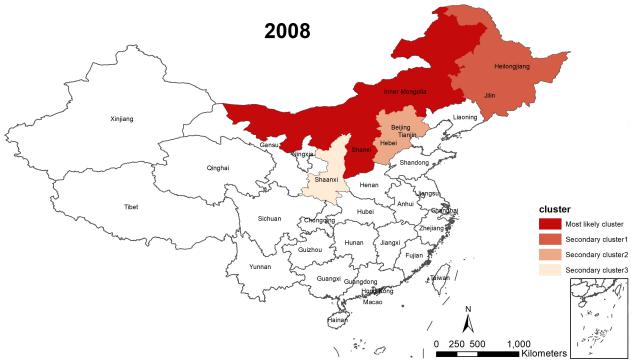

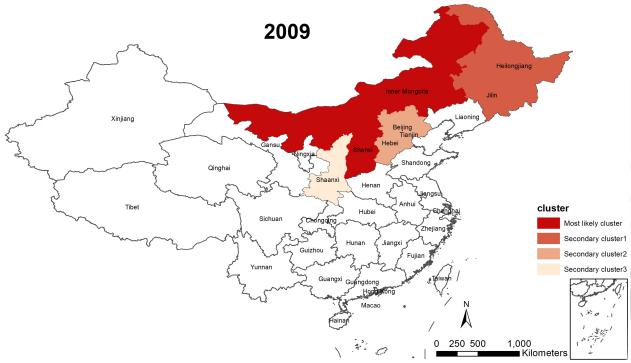

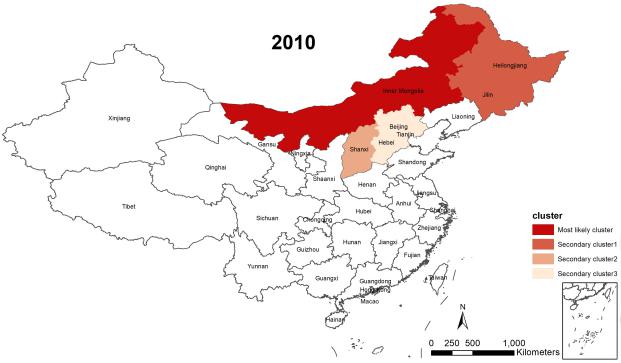

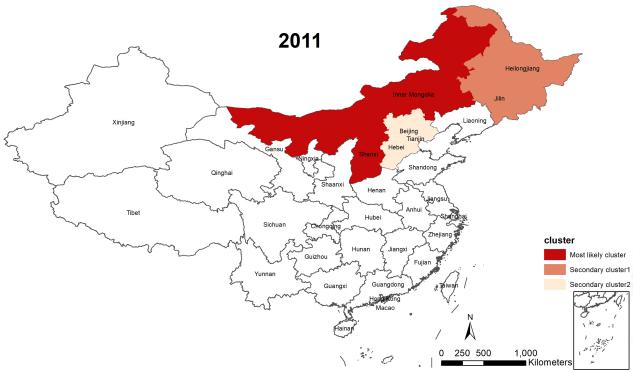

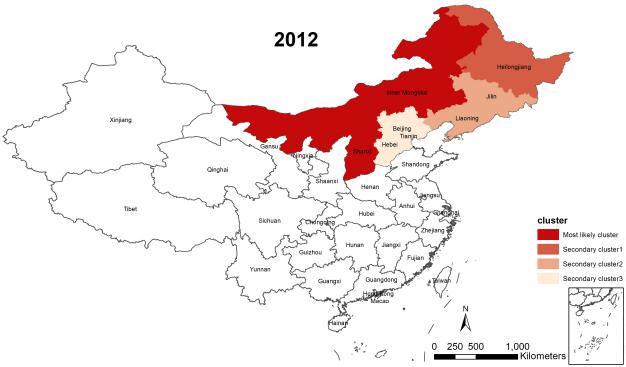

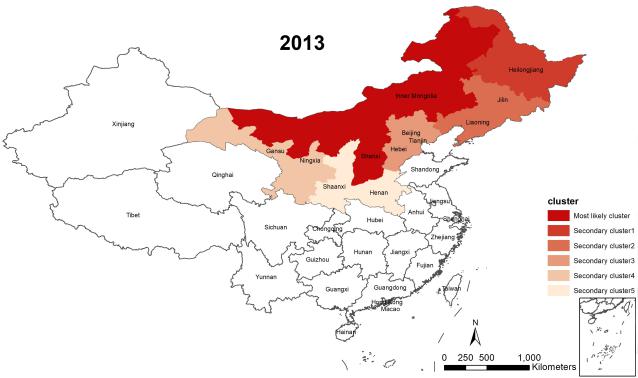

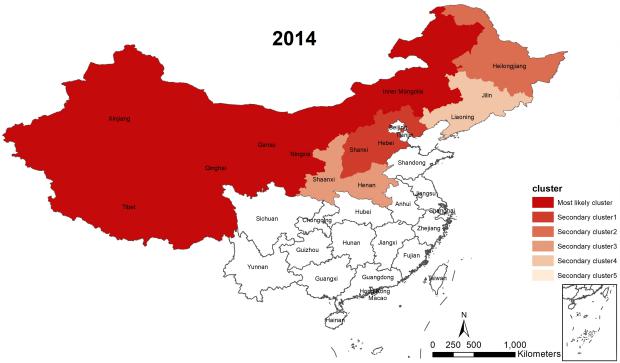

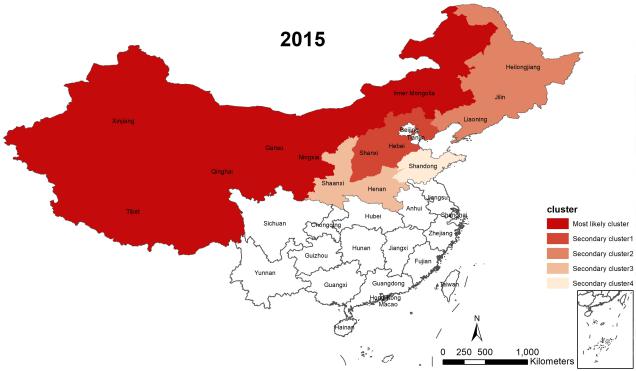

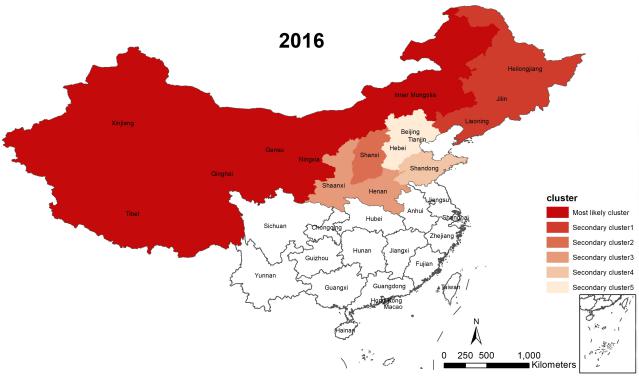

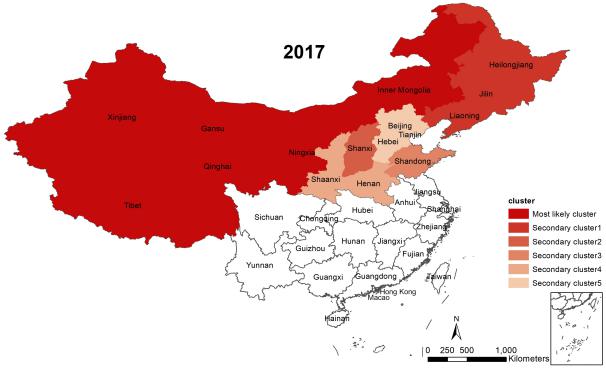


**Figure S3** Spatio-temporal distribution of high-incidence clusters between 2004 and 2017 in mainland China. Most likely clusters had spread from Inner Mongolia and Shanxi province in 2004 to Inner Mongolia, Xinjiang, Tibet, Qinghai, Gansu and Ningxia in 2017. Spatial clusters of high brucellosis incidence concentrated in northern areas of mainland China.


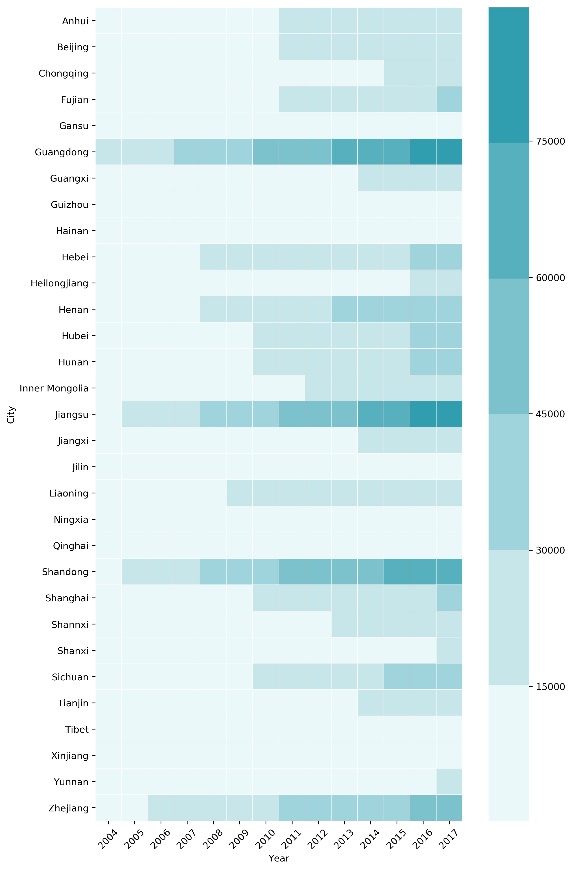

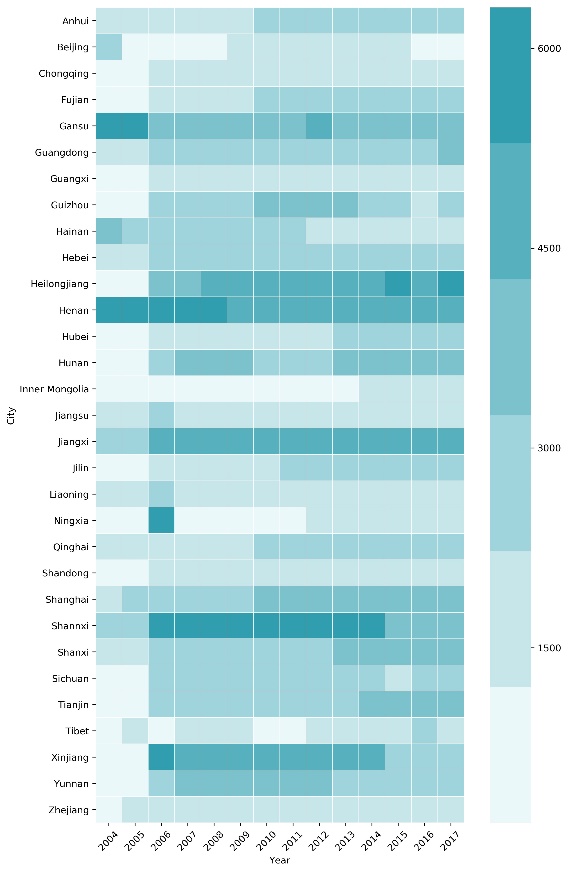


**Figure S4** GDP (left) and population density (right) provincial distribution in mainland China between 2004 and 2017. GDP in Guangdong, Jiangsu and Shandong province were higher than other provinces between 2004 and 2017. Henan, Shaanxi and Jiangxi province were more populated between 2004 and 2017 than other provinces in mainland China.


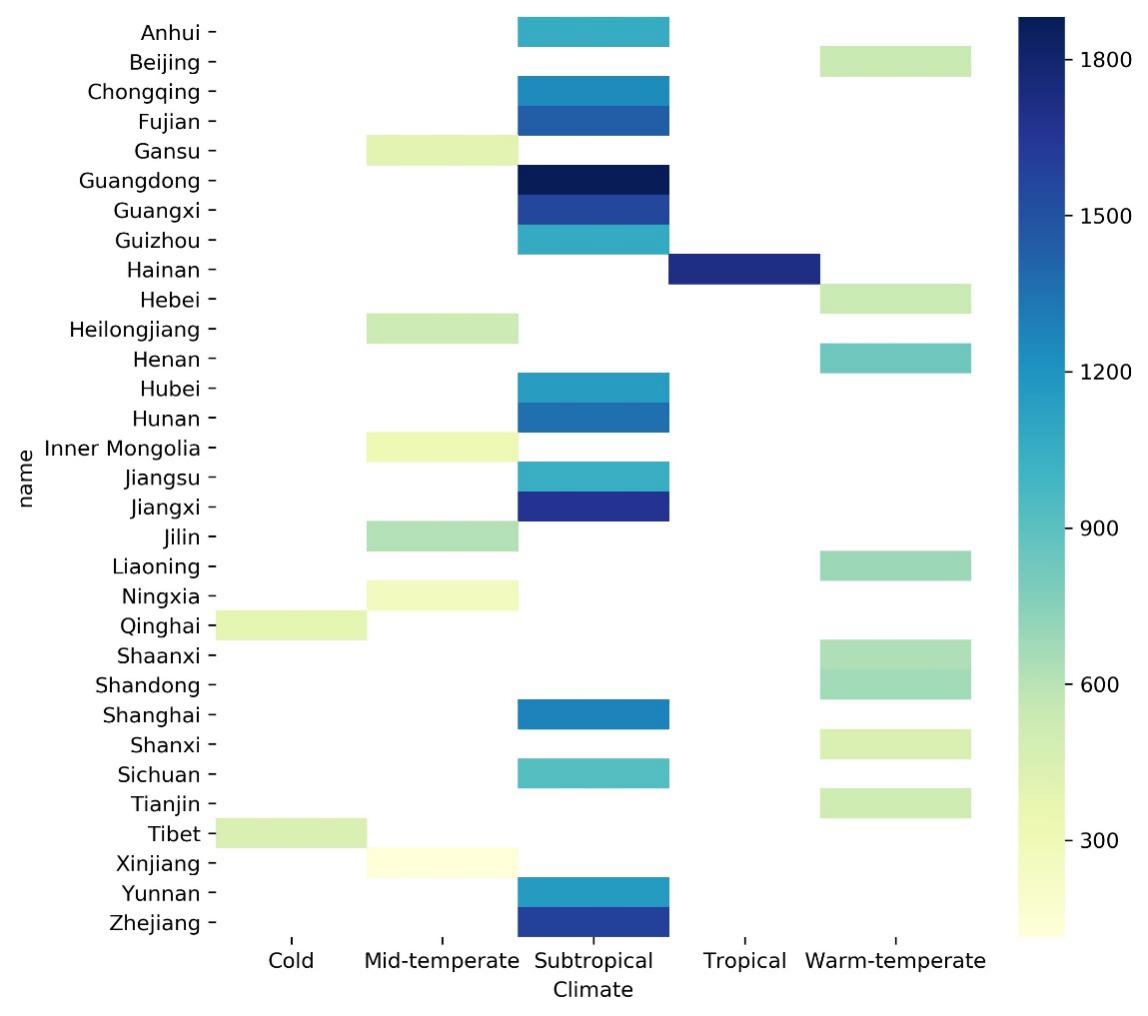
**Figure S5** Climate and Precipitation distribution in provinces of mainland China between 2004 and 2017. Subtropical areas had the most precipitation, mid-temperate and warm-temperate areas rained less. The cold areas rained the least between 2004 and 2017 among other regions in mainland China.


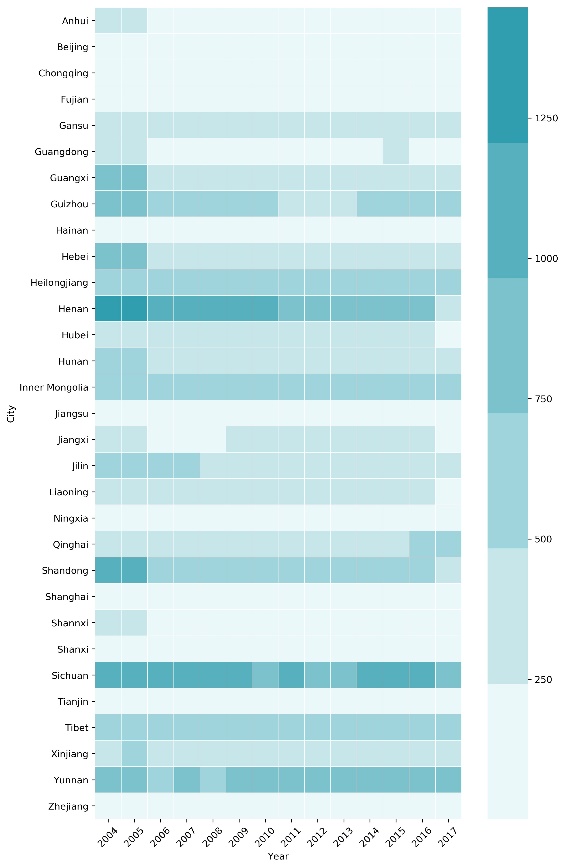

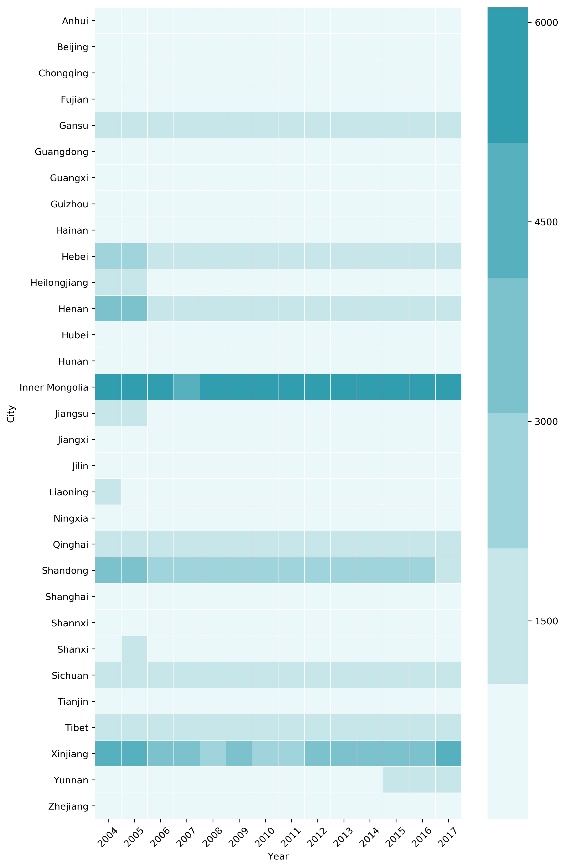

**Figure S6** Cattle stocks (left) and Sheep stocks (right) provincial distribution in mainland China between 2004 and 2017. Henan had the largest number of cattle stocks, and Inner Mongolia was had the largest average stock of sheep among other provinces between 2004 and 2017.


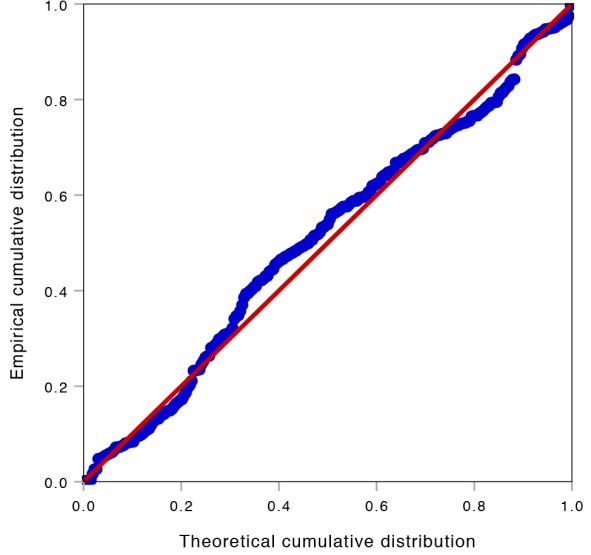

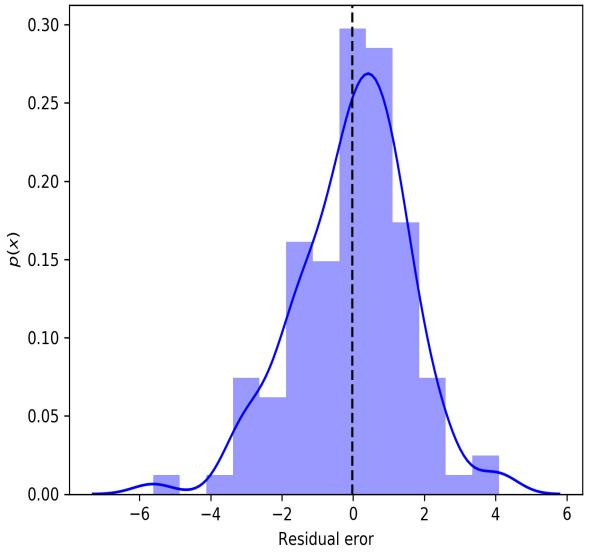


(a)

(b)


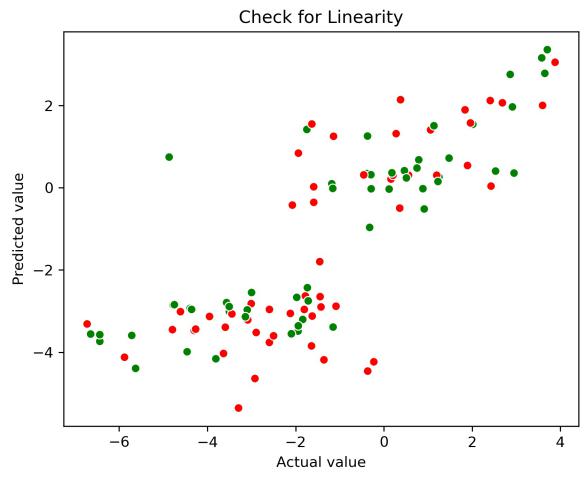


(c)

**Figure S7** Validation of multivariate linear model: (a) P-P plot, (b) Residual error and (c) linearity check. Linear regression follows the basic assumptions: variables conform to multivariate normal distribution. Thus, in P-P plot, the data points should be as much in line with y=x function line to be assumed as normally distributed (a); residual errors need to be normally distributed and the mean of residual errors should be as close to zero as possible (b); the relationship between the observed and predicted values is linear (Red dot represented the observed values in testing set, and the green dots represented the predicted values) (c).

**References**

1. Paul A. When Can You Safely Ignore Multicollinearity? 2012. *Available at:* <https://statisticalhorizons.com/multicollinearity> (Accessed 1 September 2019).

2. Lai S, Zhou H, Xiong W, Gilbert M, Huang Z, Yu J, et al. Changing epidemiology of human brucellosis, China, 1955-2014. Emerg Infect Dis. 2017;23(2):184-94.

3. Feng L, Shay DK, Jiang Y, Zhou H, Chen X, Zheng Y, et al. Influenza-associated mortality in temperate and subtropical Chinese cities, 2003-2008. Bull World Health Organ. 2012;90(4):279–288B.
